# Supplementary material for: State-level tracking of COVID-19 in the United States
Source: Nat Commun. 2020 Dec 3;11:6189. doi: 10.1038/s41467-020-19652-6 (PMC7712910; doi:10.1038/s41467-020-19652-6)
Supplement: Supplementary file 1 — Supplementary Information [file 41467_2020_19652_MOESM1_ESM.pdf]

Supplementary Information: State-level tracking of COVID-19 in the United States

H. Juliette T. Unwin[1,*,†], Swapnil Mishra[1,*], Valerie C. Bradley[2,*], Axel Gandy[3,*], Thomas A. Mellan[1], Helen Coupland[1], Jonathan Ish-Horowicz[3], Michaela A. C. Vollmer[1], Charles Whittaker[1], Sarah L. Filippi[3], Xiaoyue Xi[3], Mélodie Monod[3], Oliver Ratmann[3], Michael Hutchinson[2], Fabian Valka , Harrison Zhu[3], Iwona Hawryluk[1], Philip Milton[1], Kylie E. C. Ainslie[1], Marc Baguelin[1,4], Adhiratha Boonyasiri[5], Nick F. Brazeau[1], Lorenzo Cattarino[1], Zulma Cucunuba[1], Gina Cuomo-Dannenburg[1], Ilaria Dorigatti[1], Oliver D. Eales[1], Jeffrey W. Eaton[6], Sabine L. van Elsland[1], Richard G. FitzJohn[1], Katy A. M. Gaythorpe[1], William Green[1], Wes Hinsley[1], Benjamin Jeffrey[1], Edward Knock[1], Daniel J. Laydon[1], John Lees[1], Gemma Nedjati-Gilani[1], Pierre Nouvellet[1,7], Lucy Okell[1], Kris V. Parag[1], Igor Siveroni[1], Hayley A. Thompson[1], Patrick Walker[1], Caroline E. Walters[1], Oliver J. Watson[8,1], Lilith K. Whittles[1], Azra C. Ghani[1], Neil M. Ferguson[1], Steven Riley[1], Christl A. Donnelly[1,2], Samir Bhatt[1,*,†], and Seth Flaxman[3,*,†]

[1]MRC Centre for Global Infectious Disease Analysis, Abdul Latif Jameel Institute for Disease and Emergency Analytics (J-IDEA), Imperial College, London, UK

[2]Department of Statistics, University of Oxford, Oxford, UK

[3]Department of Mathematics, Imperial College, London, UK

[4]Department of Infectious Disease Epidemiology, London School of Hygiene and Tropical Medicine, UK

[5]NIHR Health Protection Research Unit in Healthcare Associated Infections and Antimicrobial Resistance, Imperial College London, UK

[6]MRC Centre for Global Infectious Disease Analysis, Imperial College, London, UK

[7]School of Life Sciences, University of Sussex, Brighton, UK

[8]Department of Laboratory Medicine and Pathology, Brown University, Providence, RI, USA

[*]Contributed equally

†Corresponding authors: h.unwin@imperial.ac.uk, s.bhatt@imperial.ac.uk and s.flaxman@imperial.ac.uk

Table of Contents

**Supplementary Note 1**	**Model regions**	**3**
**Supplementary Note 2**	**Ratio of reported cases to estimated infections**	**3**
**Supplementary Note 3**	**State-level R_t values**	**4**
**Supplementary Note 4**	**Model predictions for all states**	**5**
**Supplementary Note 5**	**Effect sizes**	**14**
**Supplementary Note 6**	**Forecast evaluation**	**16**
**Supplementary Note 7**	**Mobility trends**	**20**
**Supplementary Note 8**	**Mobility regression analysis**	**22**
**Supplementary Note 9**	**Comparison of model attack rates with and without interventions in the model**	**22**
**Supplementary Note 10**	**State-specific weekly effects**	**23**
**Supplementary Note 11**	**Comparison of R_t estimations**	**25**
**Supplementary Note 12**	**Median absolute percent error**	**26**
**Supplementary Note 13**	**Sensitivity analysis to infection fatality ratio**	**29**

Supplementary Note 1 Model regions

We choose 8 regions in our model, see Supplementary Figure 1. These regions are based on US Census Divisions, modified to account for coordination between groups of state, see Section 4 for more information.

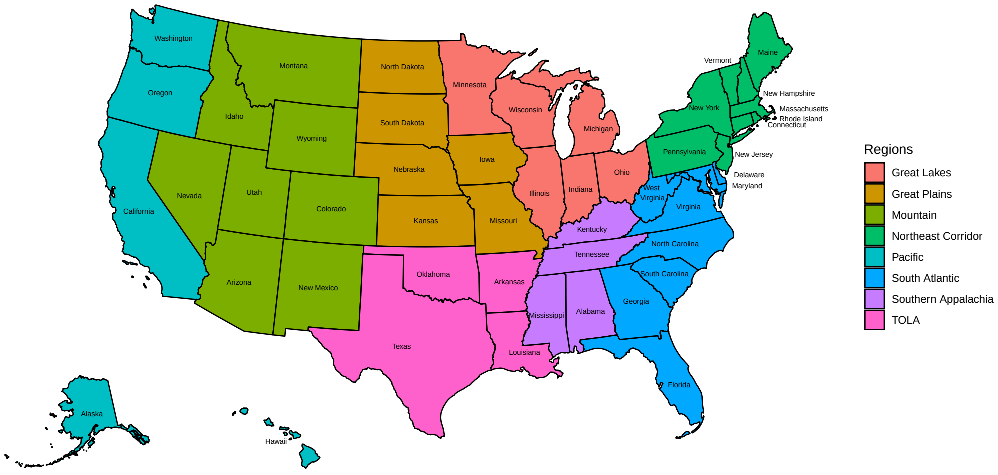

Supplementary Figure 1: **Map showing the 8 regions in our model.** The colours correspond to the regions in our models: Great Lakes - Illinois Indiana, Michigan, Minnesota, Ohio and Wisconsin; Great Plains - Iowa, Kansas, Missouri, Nebraska, North Dakota and South Dakota; Mountain - Arizona, Colorado, Idaho, Montana, Nevada, New Mexico, Utah and Wyoming; Northeast Corridor - Connecticut, Massachusetts, Maine, New Hampshire, New Jersey, New York, Pennsylvania, Rhode Island and Vermont; Pacific - Alaska, California, Hawaii, Oregon and Washington; South Atlantic - Delaware, District of Columbia, Florida, Georgia, Maryland, North Carolina, South Carolina, Virginia and West Virginia; Southern Appalachia - Alabama, Kentucky, Mississippi and Tennessee; TOLA - Arkansas, Louisiana, Oklahoma, Texas. We used a shape file from the US Census Bureau [1] for the state outlines.

Supplementary Note 2 Ratio of reported cases to estimated infections

We compare the weekly moving average of the percentage of reported cases to mean estimated infections for the time period of the model in Supplementary Figure 2. We use the same model formulation without cases here to illustrate two points. First, the percentage is small initially, which is why we do not use cases in the model until 11 May 2020. Second, sufficient tests are being done towards the end of May so they are informative to our model and we can estimate an infection ascertainment ratio, see Section 4 for further details. Percentages may be greater than 100% when our model estimates fewer cases than were reported. This occurs in states with little data.

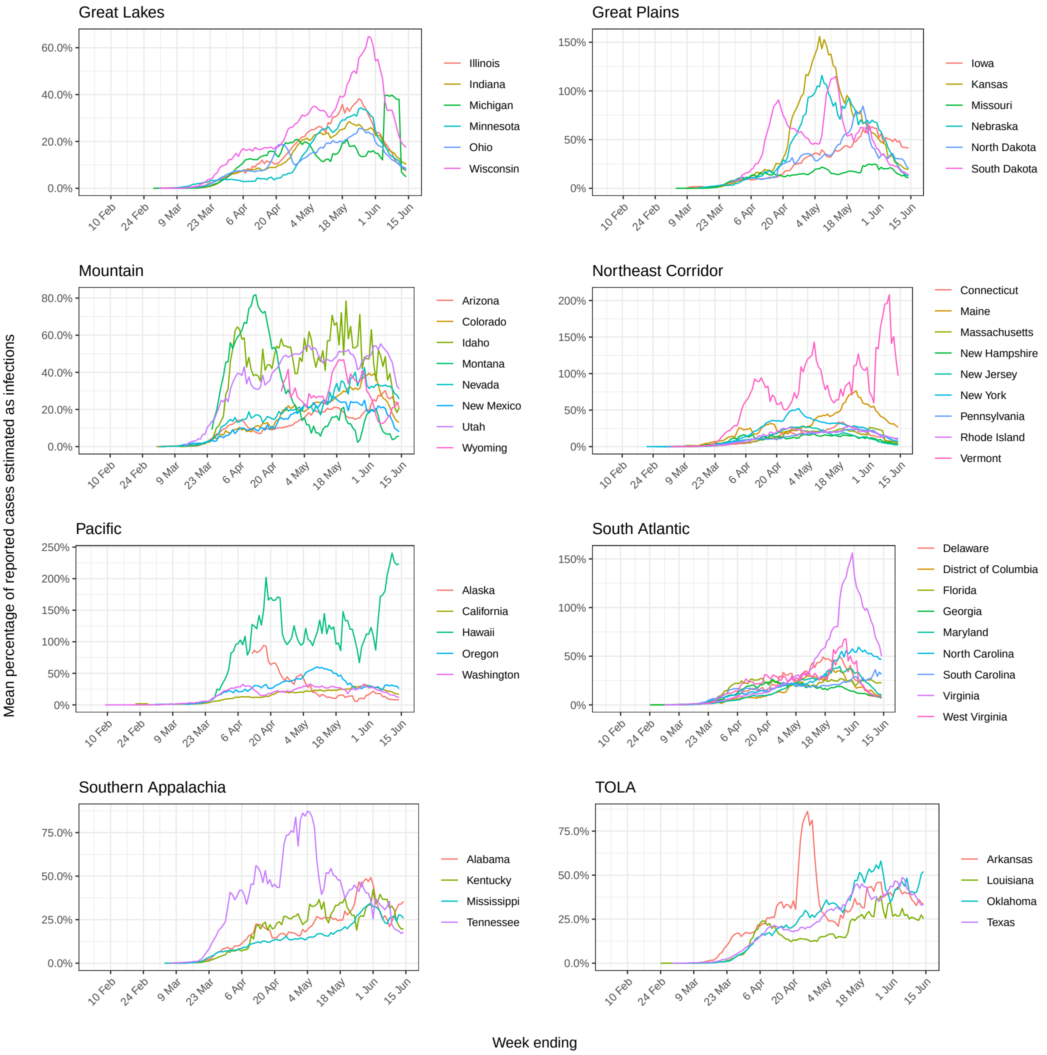

Supplementary Figure 2: **Weekly moving average of the percentage of reported cases to mean estimated infections for the time period of the model.** The colours correspond to the States in each region subfigure.

Supplementary Note 3 State-level R_t values

Supplementary Figure 3 shows the value of R_t at two points during the epidemic. The first time point is for the week centered on the date on which each state implemented any emergency decree order such as State of Emergency, Public Health Emergency, and Public Health Disaster declarations. The second is for the week ending on 01 June 2020.

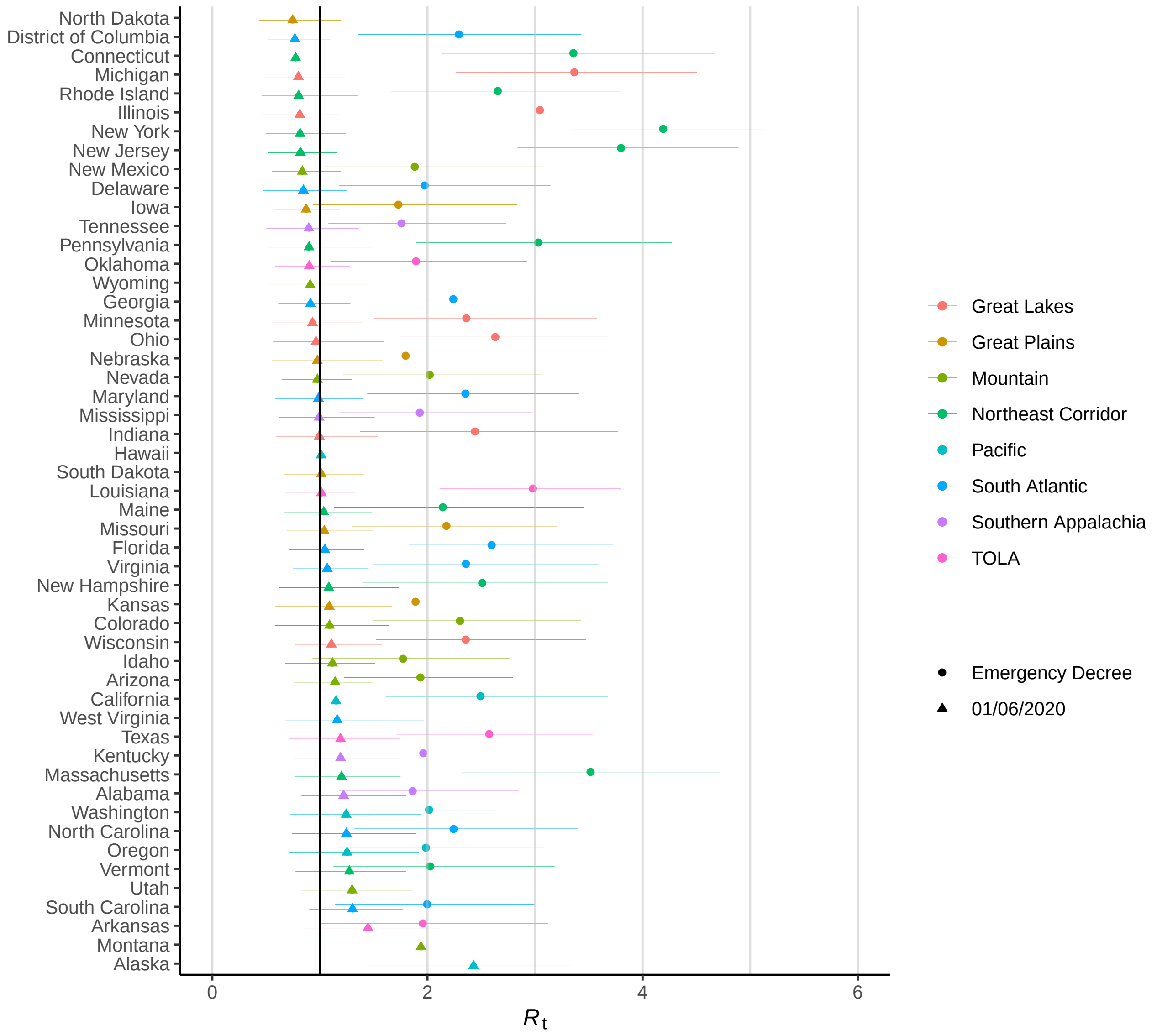

Supplementary Figure 3: **State-level estimates of $R_{t=EmergencyDecree}$ and the average R_t over the week ending 01 June 2020.** The colours indicate regional grouping as shown in Supplementary Figure 1. We do not include estimates of $R_{t=EmergencyDecree}$ for Alaska, Hawaii, Montana, North Dakota, South Dakota, Utah, West Virgina and Wyoming as Emergency Decree was declared in these states before we start modelling these states. The error bars denote the 95% credible intervals and n = 105,006 deaths across the 50 states and the District of Columbia up until 1 June and 479,422 cases from 11 May to 1 June.

Supplementary Note 4 Model predictions for all states

State-level estimates of infections, deaths and R_t. Left: daily number of deaths, brown bars are reported deaths, blue bands are predicted deaths, dark blue 50% credible interval (CI), light blue 95% CI. Middle: daily number of infections, brown bars are reported infections, blue bands are predicted infections, CIs are same as left. The number of daily infections estimated by our model drops immediately after an intervention, as we assume that all infected people become immediately less infectious through the intervention. Afterwards, if the R_t is above 1, the number of infections will start growing again. Right: time-varying reproduction number R_t dark green 50% CI,

light green 95% CI. Icons are interventions shown at the time they occurred.

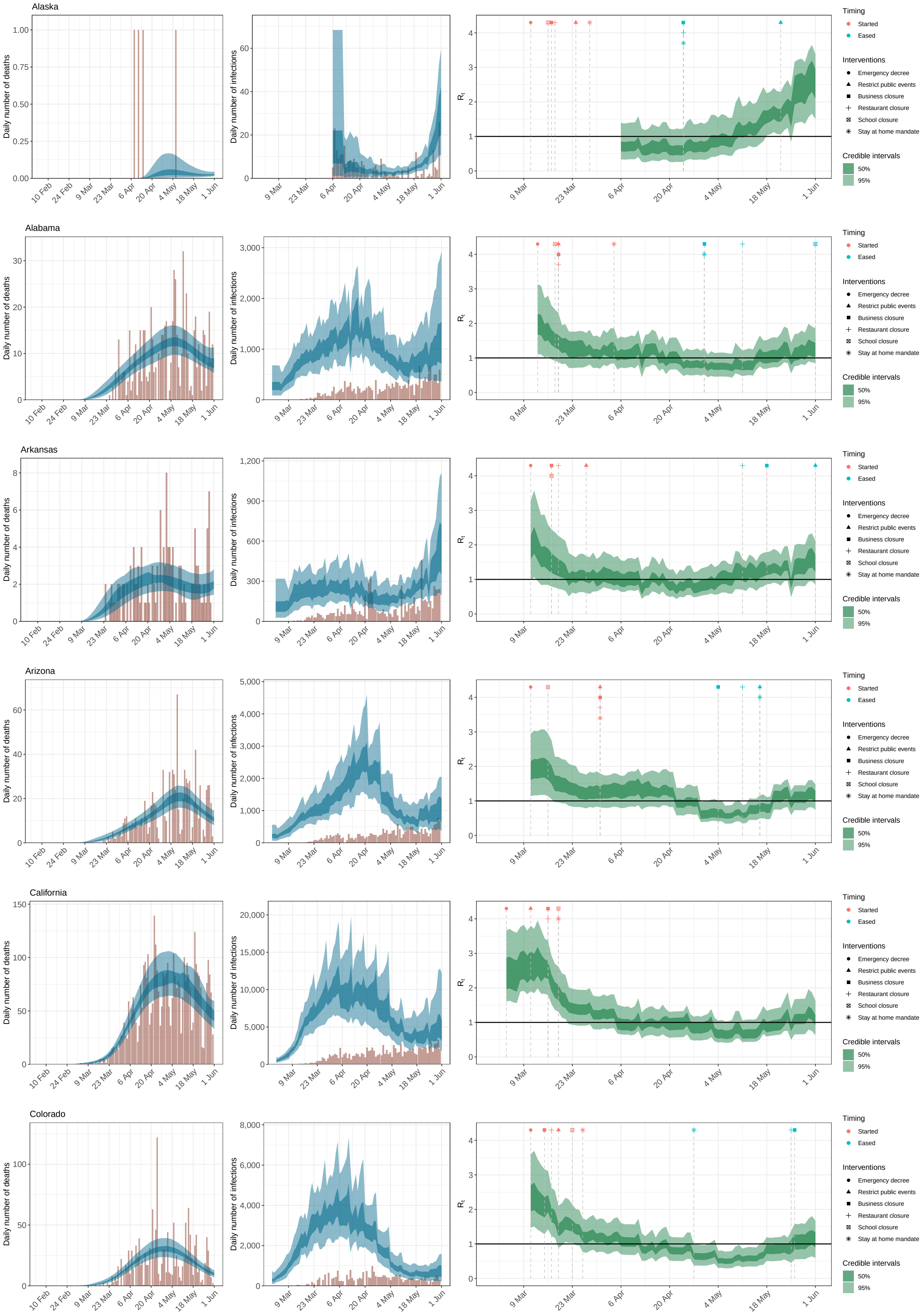

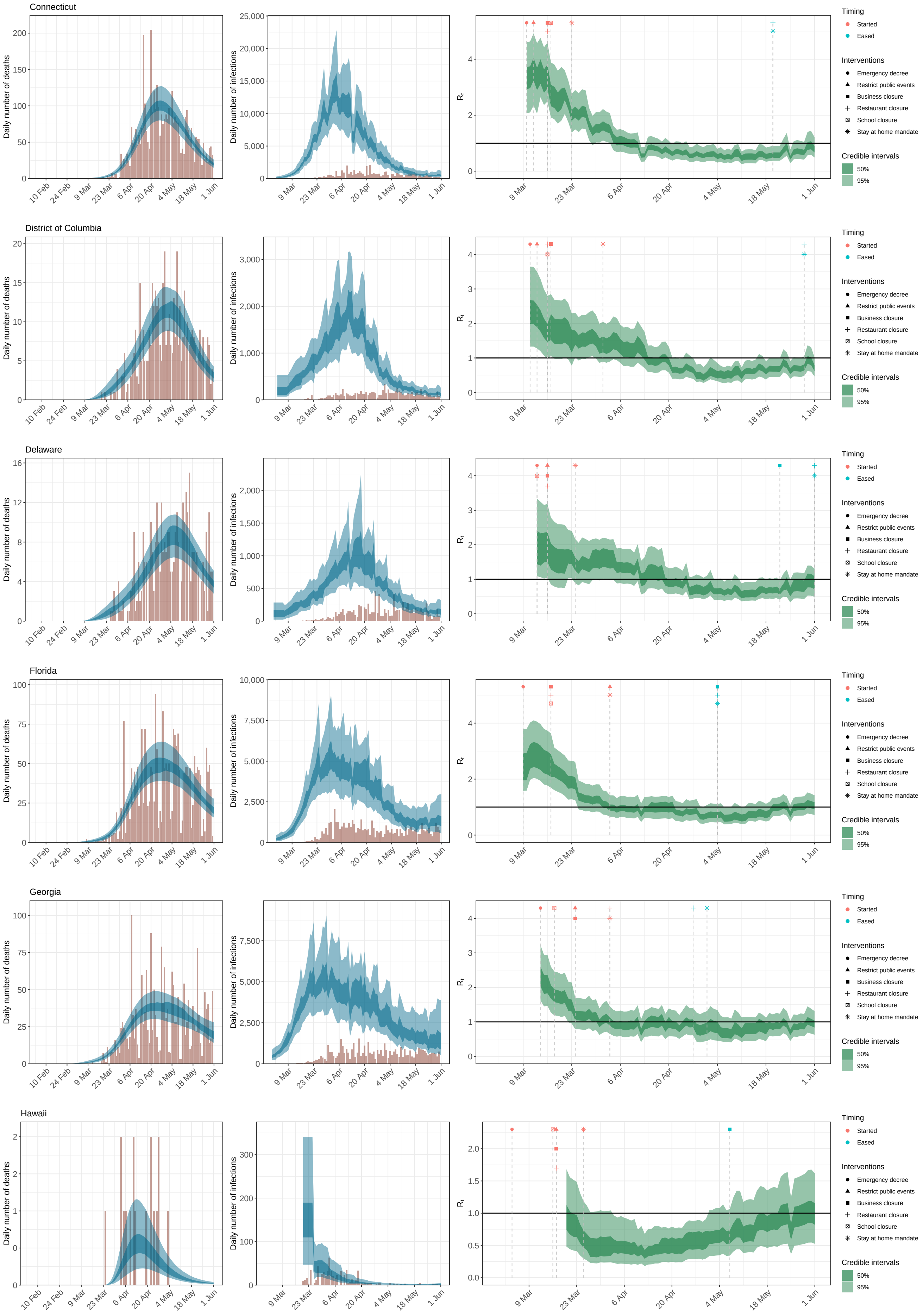

Connecticut
Daily number of deaths
Daily number of infections
R_t
10 Feb
24 Feb
9 Mar
23 Mar
6 Apr
20 Apr
4 May
18 May
1 Jun
Timing
Started
Eased
Interventions
Emergency decree
Restrict public events
Business closure
Restaurant closure
School closure
Stay at home mandate
Credible intervals
50%
95%
District of Columbia
Delaware
Florida
Georgia
Hawaii

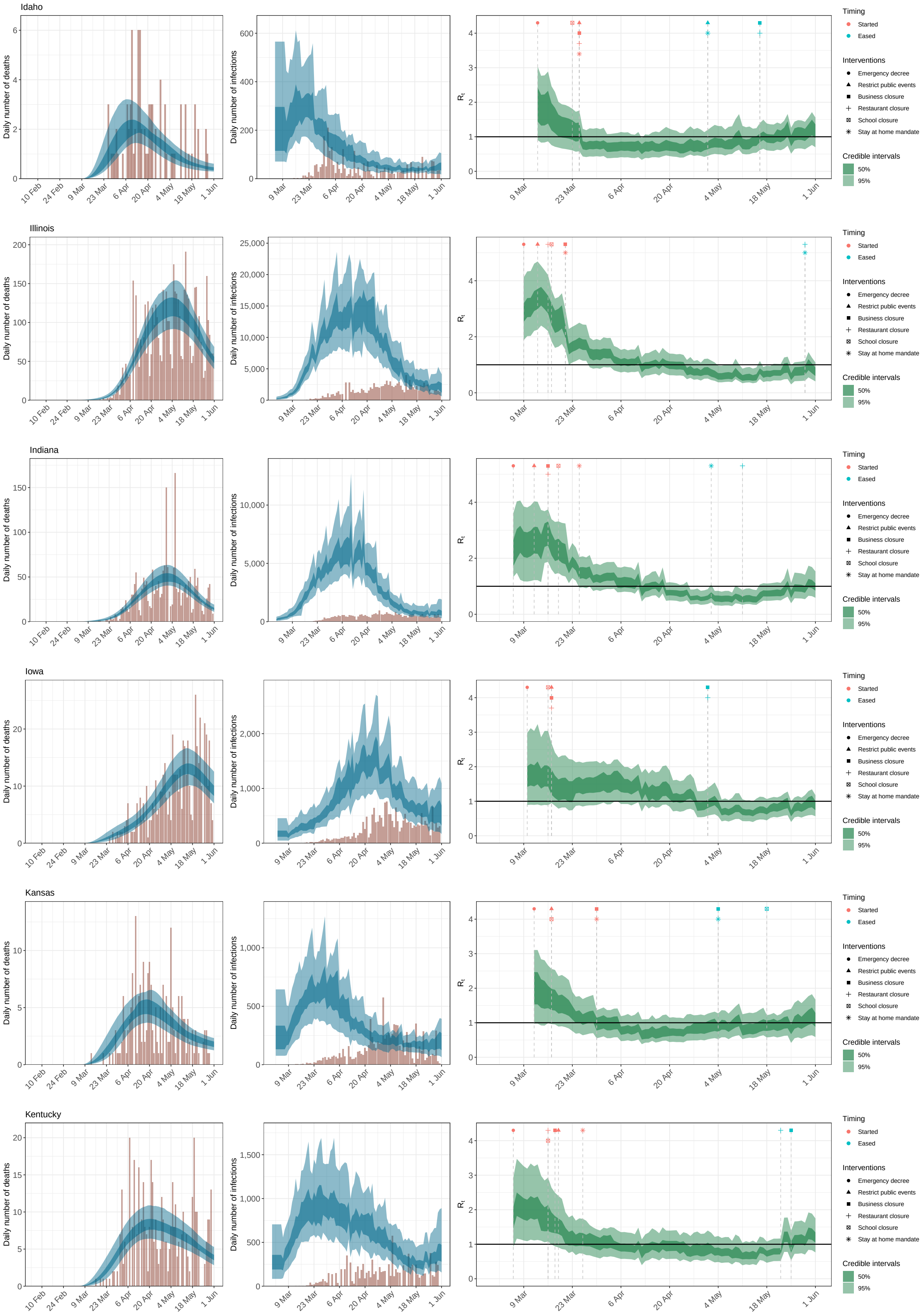
Idaho
Illinois
Indiana
Iowa
Kansas
Kentucky
Daily number of deaths
Daily number of infections
R_t
10 Feb
24 Feb
9 Mar
23 Mar
6 Apr
20 Apr
4 May
18 May
1 Jun
Timing
Started
Eased
Interventions
Emergency decree
Restrict public events
Business closure
Restaurant closure
School closure
Stay at home mandate
Credible intervals
50%
95%

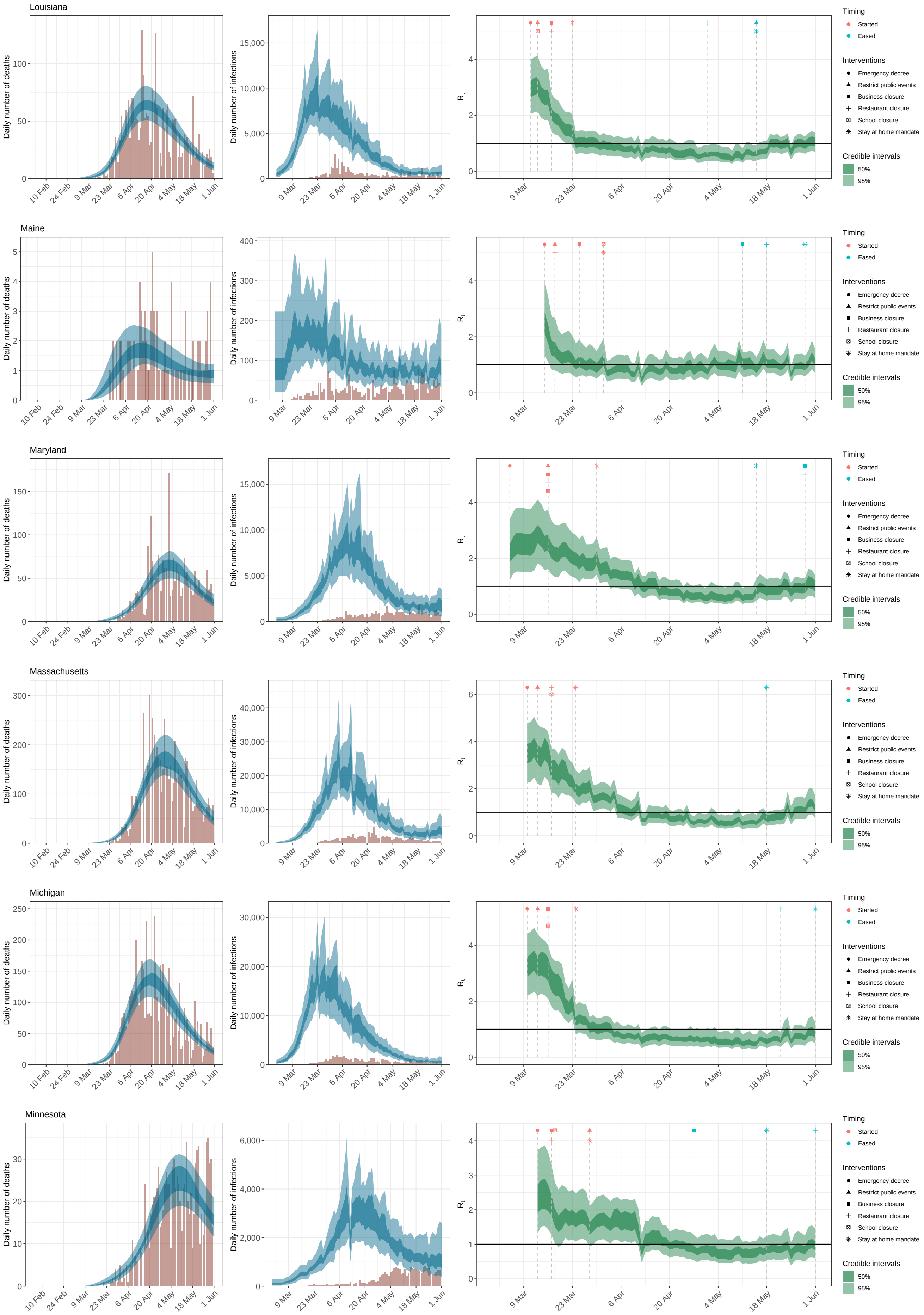
Louisiana
Maine
Maryland
Massachusetts
Michigan
Minnesota
Daily number of deaths
Daily number of infections
R_t
10 Feb
24 Feb
9 Mar
23 Mar
6 Apr
20 Apr
4 May
18 May
1 Jun
Timing
Started
Eased
Interventions
Emergency decree
Restrict public events
Business closure
Restaurant closure
School closure
Stay at home mandate
Credible intervals
50%
95%

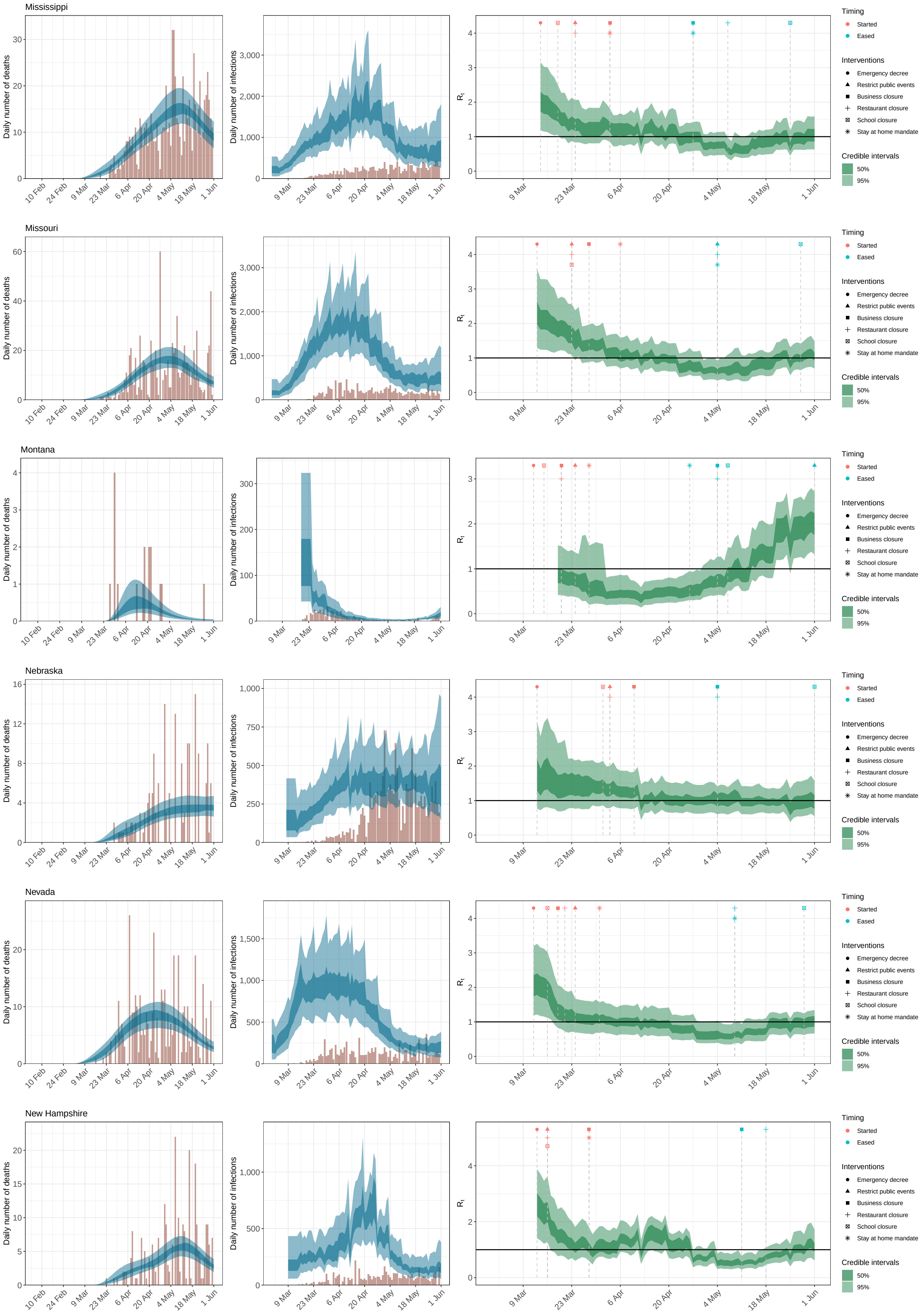
Mississippi
Missouri
Montana
Nebraska
Nevada
New Hampshire
Daily number of deaths
Daily number of infections
R_t
10 Feb
24 Feb
9 Mar
23 Mar
6 Apr
20 Apr
4 May
18 May
1 Jun
Timing
Started
Eased
Interventions
Emergency decree
Restrict public events
Business closure
Restaurant closure
School closure
Stay at home mandate
Credible intervals
50%
95%

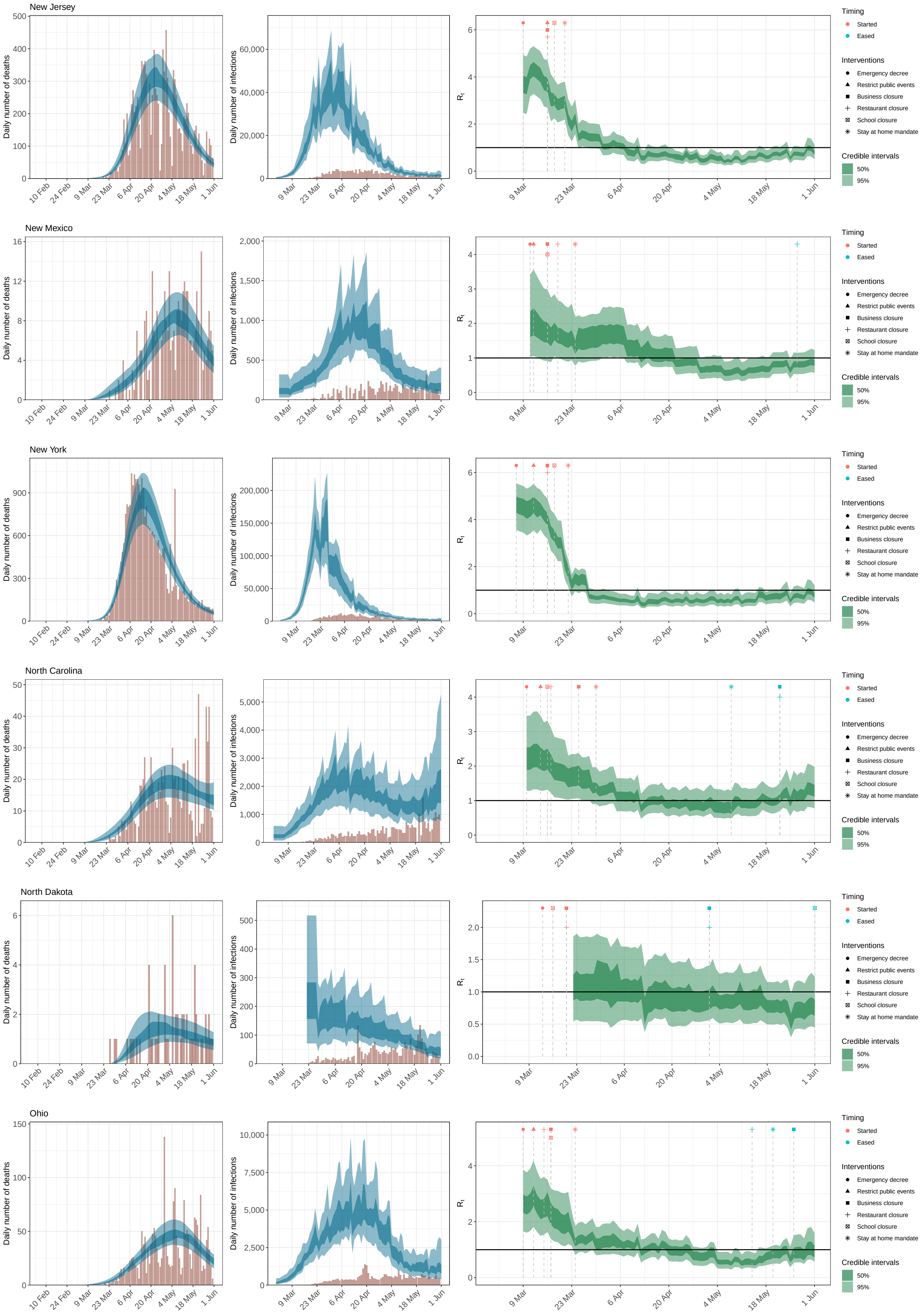
New Jersey
New Mexico
New York
North Carolina
North Dakota
Ohio
Daily number of deaths
Daily number of infections
R_t
10 Feb
24 Feb
9 Mar
23 Mar
6 Apr
20 Apr
4 May
18 May
1 Jun
Timing
Started
Eased
Interventions
Emergency decree
Restrict public events
Business closure
Restaurant closure
School closure
Stay at home mandate
Credible intervals
50%
95%

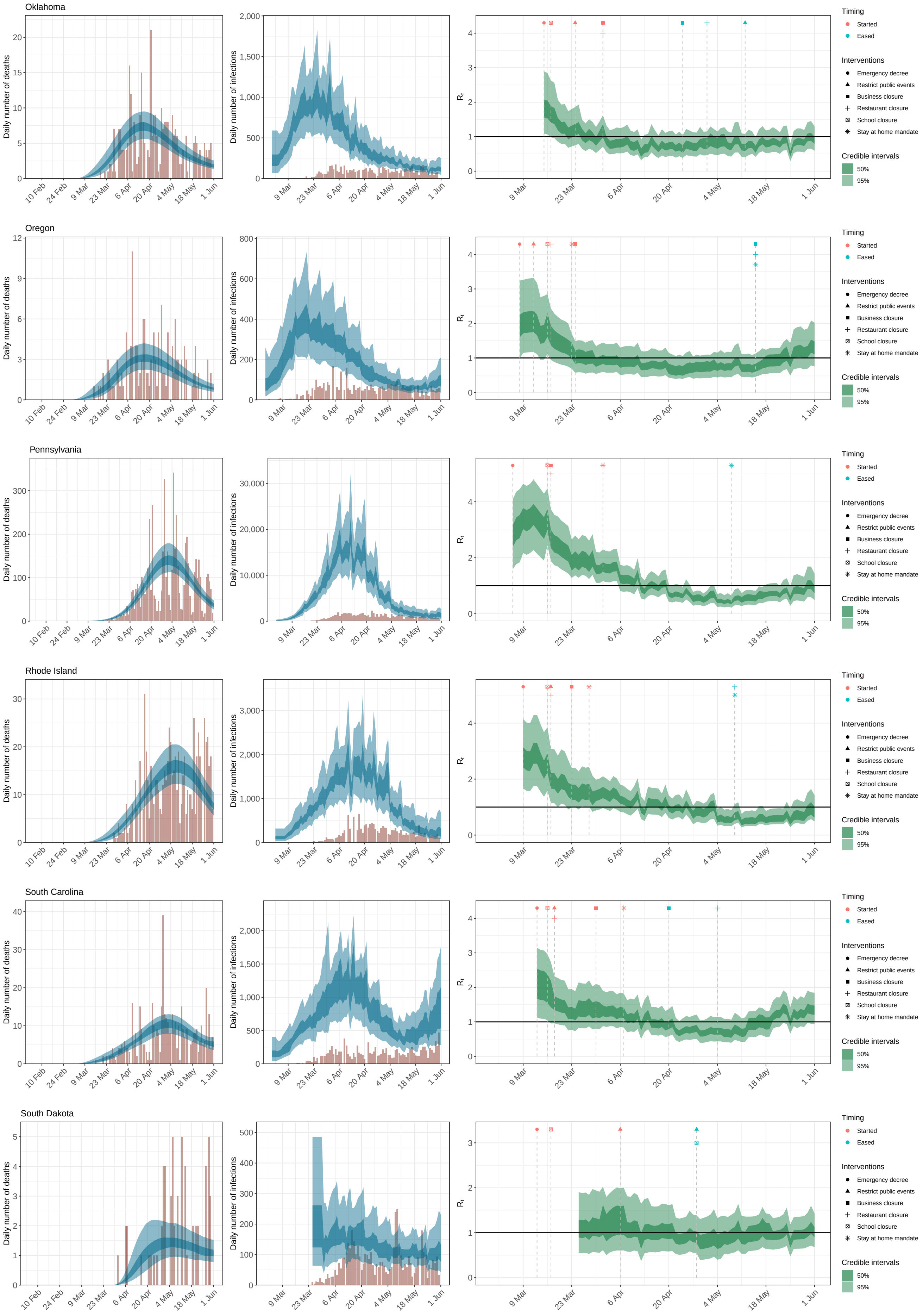
Oklahoma
Oregon
Pennsylvania
Rhode Island
South Carolina
South Dakota
Daily number of deaths
Daily number of infections
R_t
10 Feb
24 Feb
9 Mar
23 Mar
6 Apr
20 Apr
4 May
18 May
1 Jun
Timing
Started
Eased
Interventions
Emergency decree
Restrict public events
Business closure
Restaurant closure
School closure
Stay at home mandate
Credible intervals
50%
95%

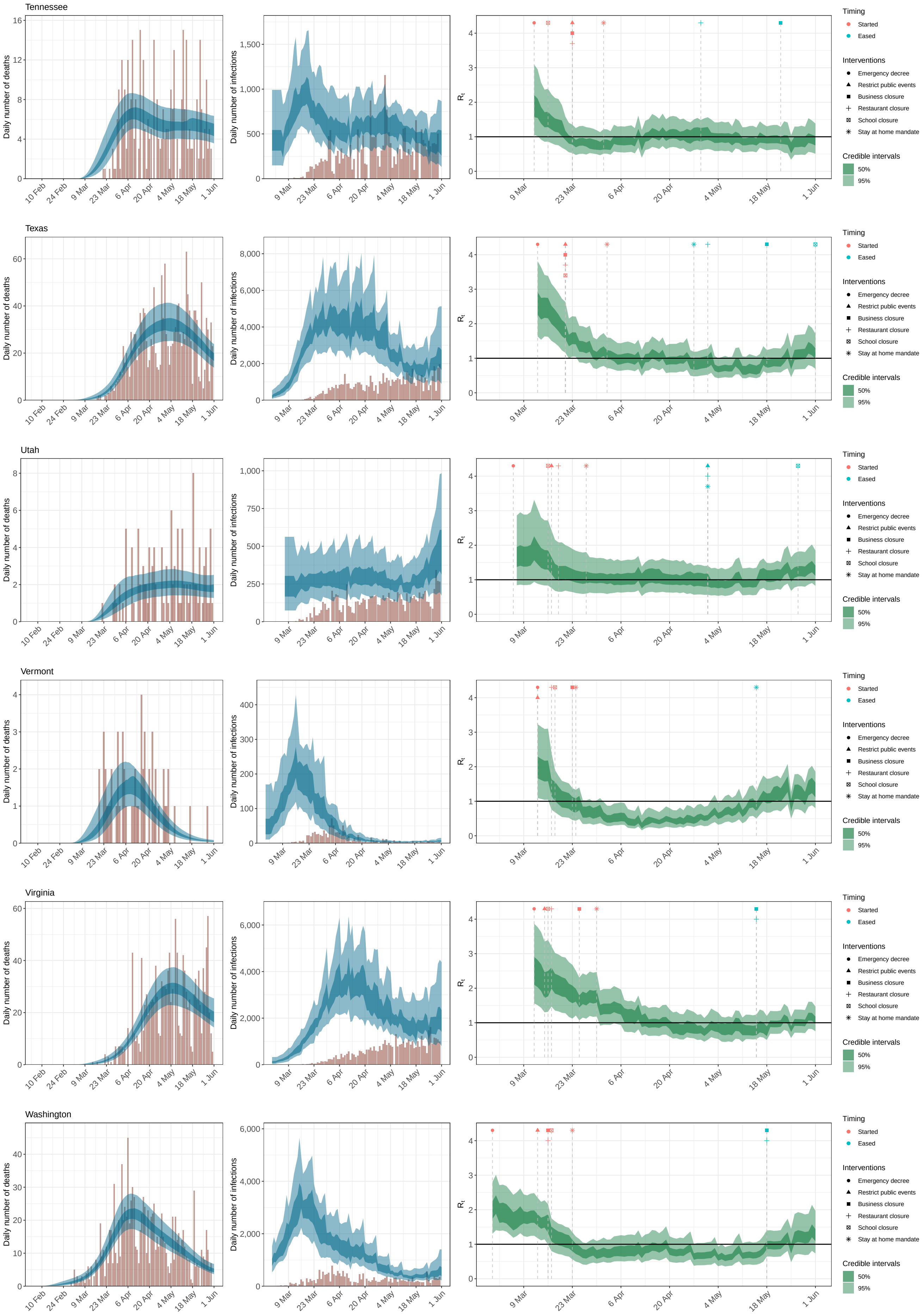
Tennessee
Texas
Utah
Vermont
Virginia
Washington
Daily number of deaths
Daily number of infections
R_t
10 Feb
24 Feb
9 Mar
23 Mar
6 Apr
20 Apr
4 May
18 May
1 Jun
Timing
Started
Eased
Interventions
Emergency decree
Restrict public events
Business closure
Restaurant closure
School closure
Stay at home mandate
Credible intervals
50%
95%

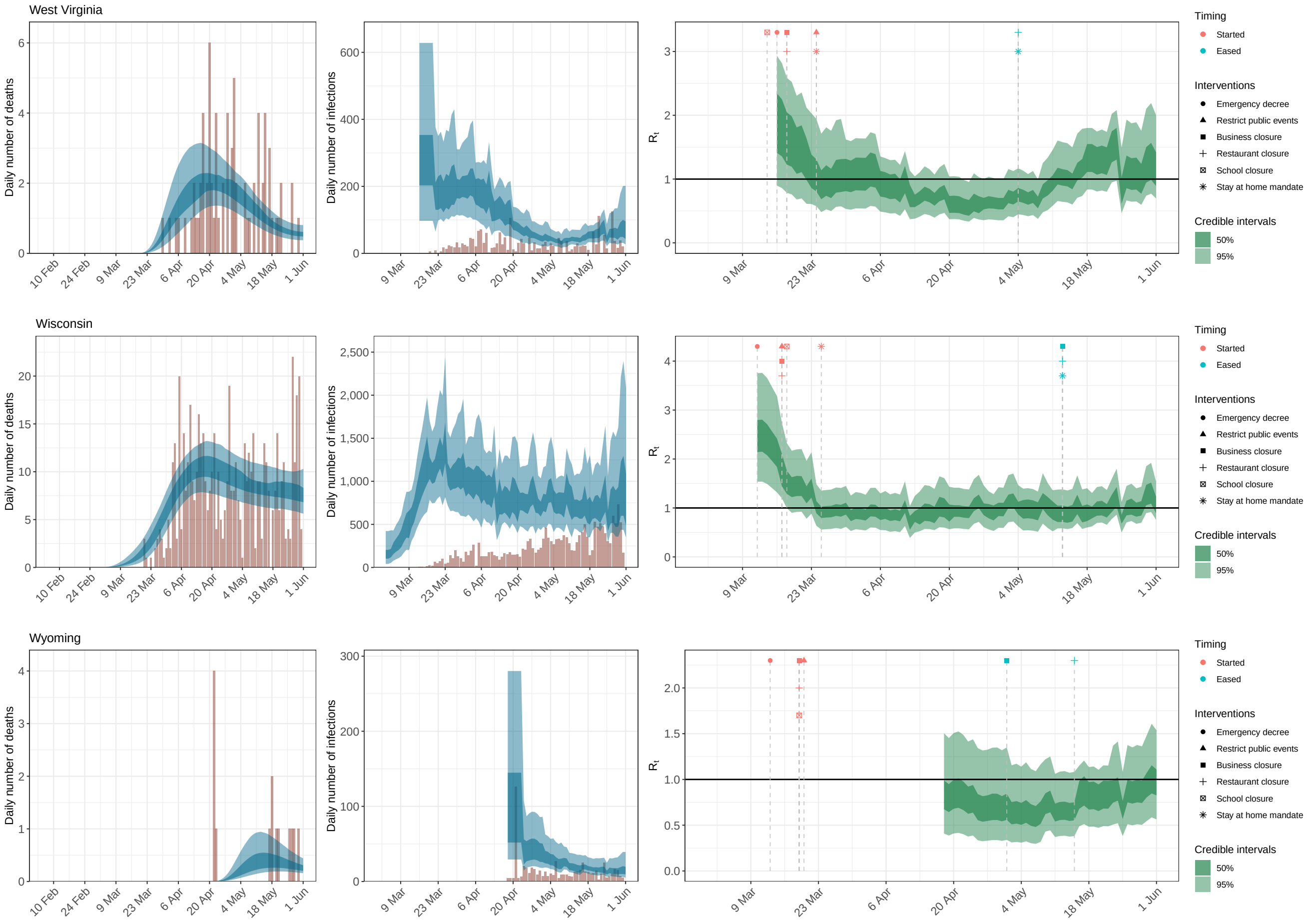

Supplementary Note 5 Effect sizes

We plot estimates of the posterior mean effect sizes and 95% credible intervals for the regional and state level covariates. The relative percentage reduction in R_t metric is interpreted as follows: the larger the percentage, the more R_t decreases, meaning the disease spreads less; a 100% relative reduction ends disease transmissibility entirely. The smaller the percentage, the less effect the covariate has on transmissibility. A 0% relative reduction has no effect on R_t and thus no effect on the transmissibility of the disease, while a negative percent reduction implies an increase in transmissibility.

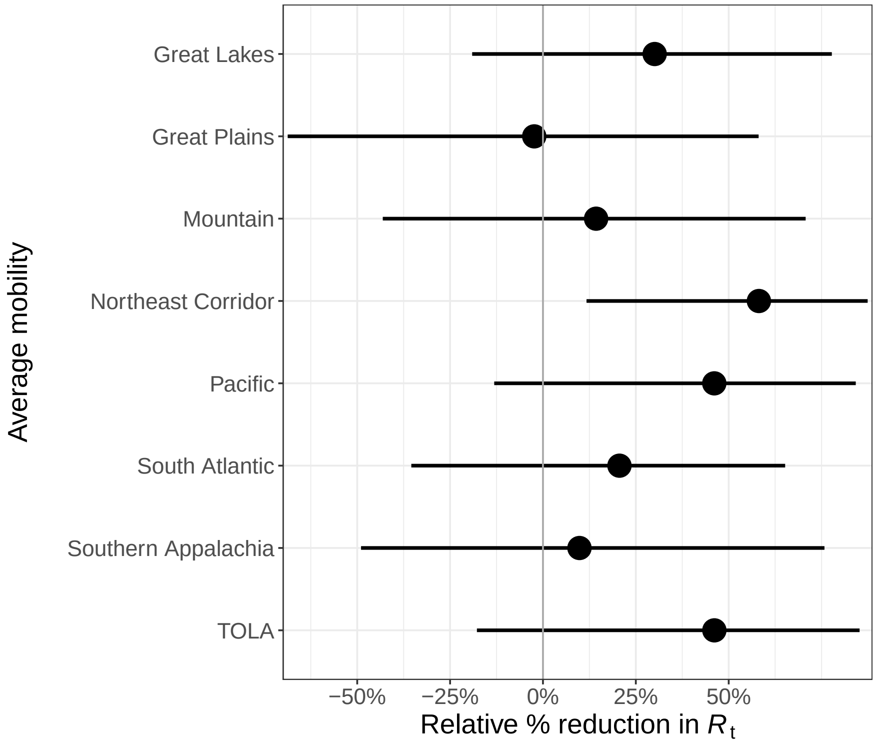

Supplementary Figure 4: **Regional average mobility covariate effect size plots assuming mobility stopped entirely (100% reduction in average mobility).** The error bars show 95% credible intervals and the dots show the mean estimate. The sample size n = 105,006 deaths across the 50 states and the District of Columbia up until 1 June and 479,422 cases from 11 May to 1 June.

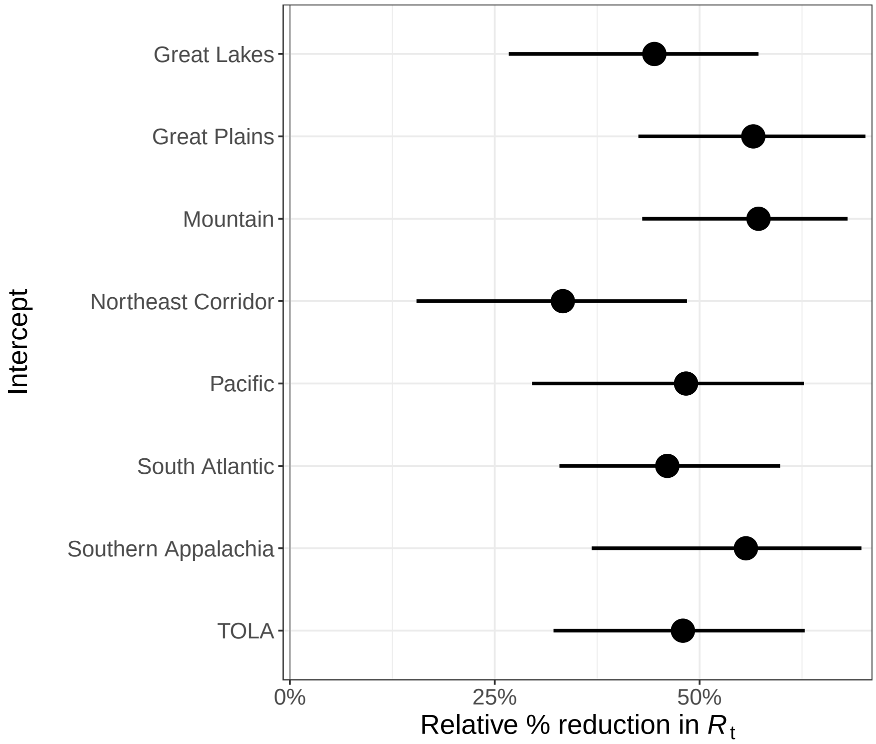

Supplementary Figure 5: **Regional intercept covariate effect size plots assuming mobility stopped entirely (100% reduction in average mobility).** The error bars show 95% credible intervals and the dots show the mean estimate. The sample size n = 105,006 deaths across the 50 states and the District of Columbia up until 1 June and 479,422 cases from 11 May to 1 June.

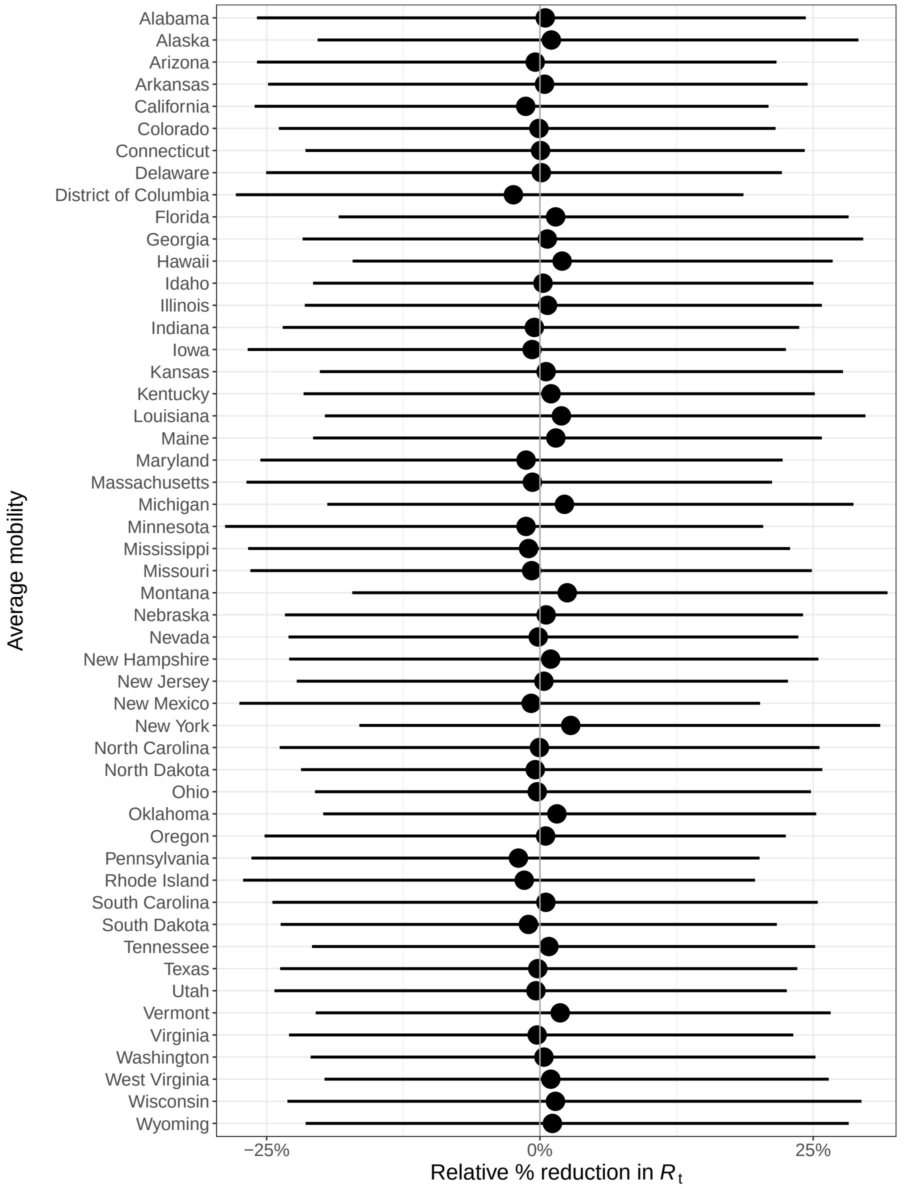

Supplementary Figure 6: **State-level covariate effect size plots assuming mobility stopped entirely (100% reduction in average mobility).** The error bars show 95% credible intervals and the dots show the mean estimate. The sample size n = 105,006 deaths across the 50 states and the District of Columbia up until 1 June and 479,422 cases from 11 May to 1 June.

Supplementary Note 6 Forecast evaluation

We evaluate three-week ahead model forecasts (days $t \in \{T+1, \ldots, T+21\}$) for our model using two metrics. The first metric is the mean absolute error (MAE), which is given by

$$\mathrm{MAE}_{t,m} = \frac{1}{S} \sum_{s=1}^{S} \left| \widehat{y}_{t,m}^{s} - y_{t,m} \right| , \qquad t = T+1, \ldots, T+21 \, , \tag{1}$$

where $\widehat{y}_{t,m}^{1}, \ldots, \widehat{y}_{t,m}^{S}$ are S posterior predictive samples of daily deaths on day t in country m and $y_{t,m}$ is the observed value of the corresponding quantity. The continuous ranked probability score (CRPS) is a generalisation

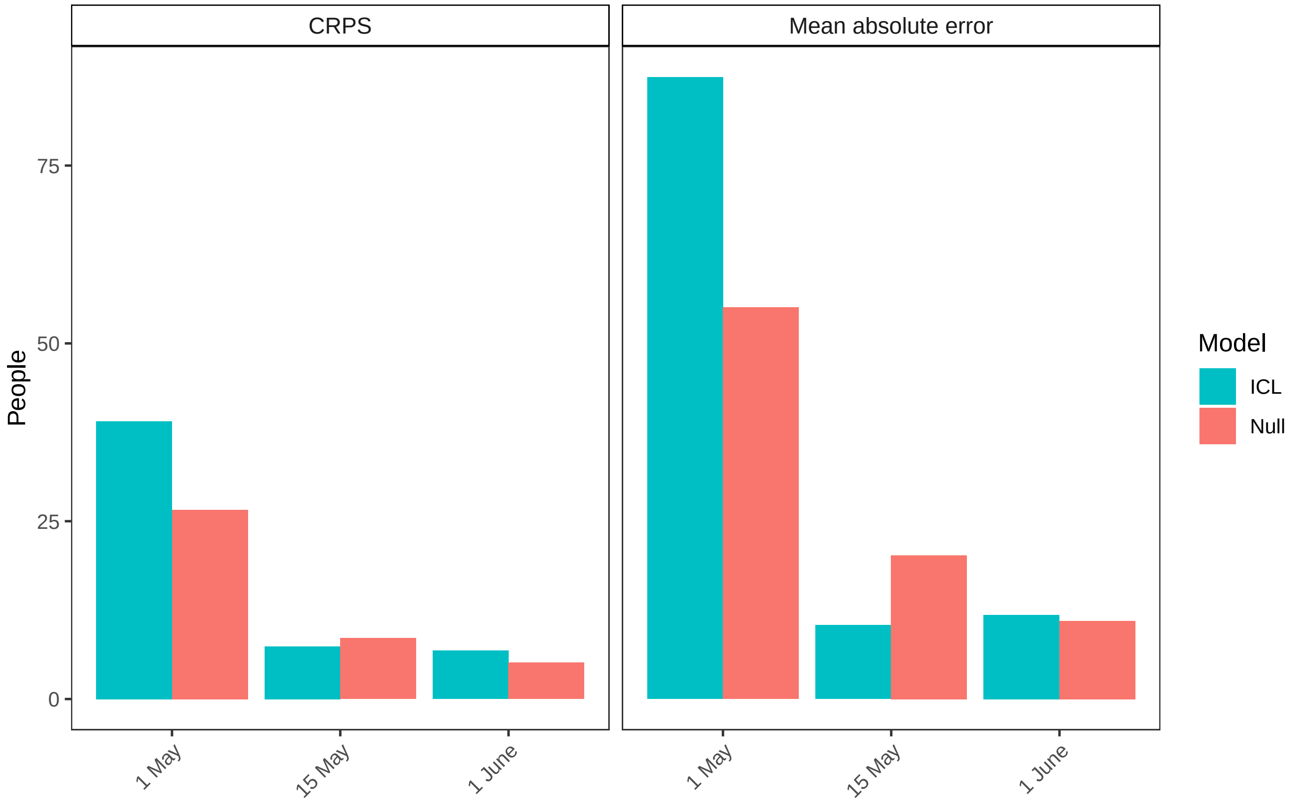

Supplementary Figure 7: **MAE and CRPS 21 day forecast estimates for three models fit until 1 May, 15 May and 1 June.** The blue ICL model refers to our model and the coral bars refer to the null model.

of MAE to probabilistic forecasts and can be estimated using Gneiting and Raftery [2]

$$\mathrm{CRPS}_{t,m} = \frac{1}{S}\sum_{s=1}^{S} |\widehat{y}^{s}_{t,m} - y_{t,m}| - \frac{1}{2S^2}\sum_{j=1}^{S}\sum_{k=1}^{S} |\widehat{y}^{j}_{t,m} - \widehat{y}^{k}_{t,m}|, \qquad t = T+1, \ldots, T+21\,. \tag{2}$$

Supplementary Figure 7 shows the mean MAE and CRPS for three different forecast periods across all time points and states. In addition to the forecast results shown in Section 2.4, we also fit our model up to 1 May and 15 May and show the error in our 21 day forecasts. We compare these forecasts to a simple log-linear "null" model fit to the 31 days of death data prior to the end dates of our model runs (1 May, 15 May and 1 June) to evaluate our predictions. We find the MAE and CRPS error were different for models fit up to different dates. Our model fit to 1 May performs worse than the "null" model because this model only includes deaths (we only include cases in our model after 11 May, see Supplementary Text 2. Our model performs similarly (1 June) or better than the null model (15 May) in both MAE and CRPS. We include a state-level break down of MAE in Supplementary Figure 8 and CRPS in Supplementary Figure 9.

The forecasts were also evaluated using the mean coverage of their 95% and 50% credible intervals. If model uncertainty is well-calibrated then the observed quantities will fall outside of the 95% credible intervals 5% of the time and 50% of the time for the 50% credible intervals. Recent work has highlighted that other prominent models do not meet this criterion, which suggests that their uncertainty estimates are not well-calibrated [3]. The mean coverage of the 95% credible interval in a time period starting at time t_0 with length L is given by

$$\frac{1}{L}\sum_{t=t_0+1}^{t_0+1+L} 1\left(y_{t,m} \in \left[p_{2.5}(\{\widehat{y}^{s}_{t,m}\}_{s=1}^{S}), p_{97.5}(\{\widehat{y}^{s}_{t,m}\}_{s=1}^{S})\right]\right), \tag{3}$$

where $1(\cdot)$ is the indicator function and $p_z(\{\widehat{y}^{s}_{t,m}\}_{s=1}^{S})$ is the z-th percentile of the samples on day t in country m.

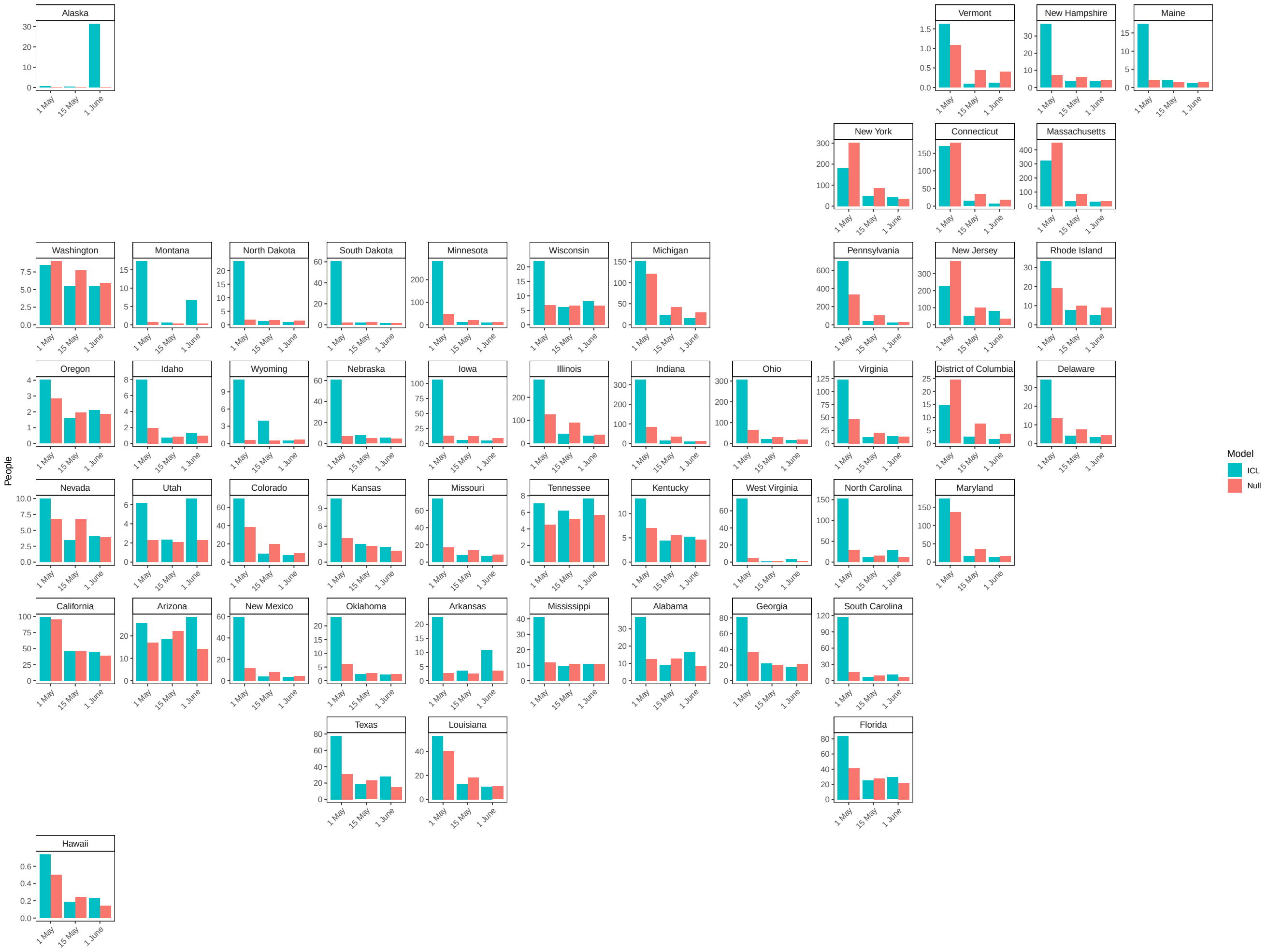

Supplementary Figure 8: **State level 21 day forecast mean absolute error estimates for three models fit until 1 May, 15 May and 1 June.** The blue ICL model refers to our model and the coral bars refer to the null model.

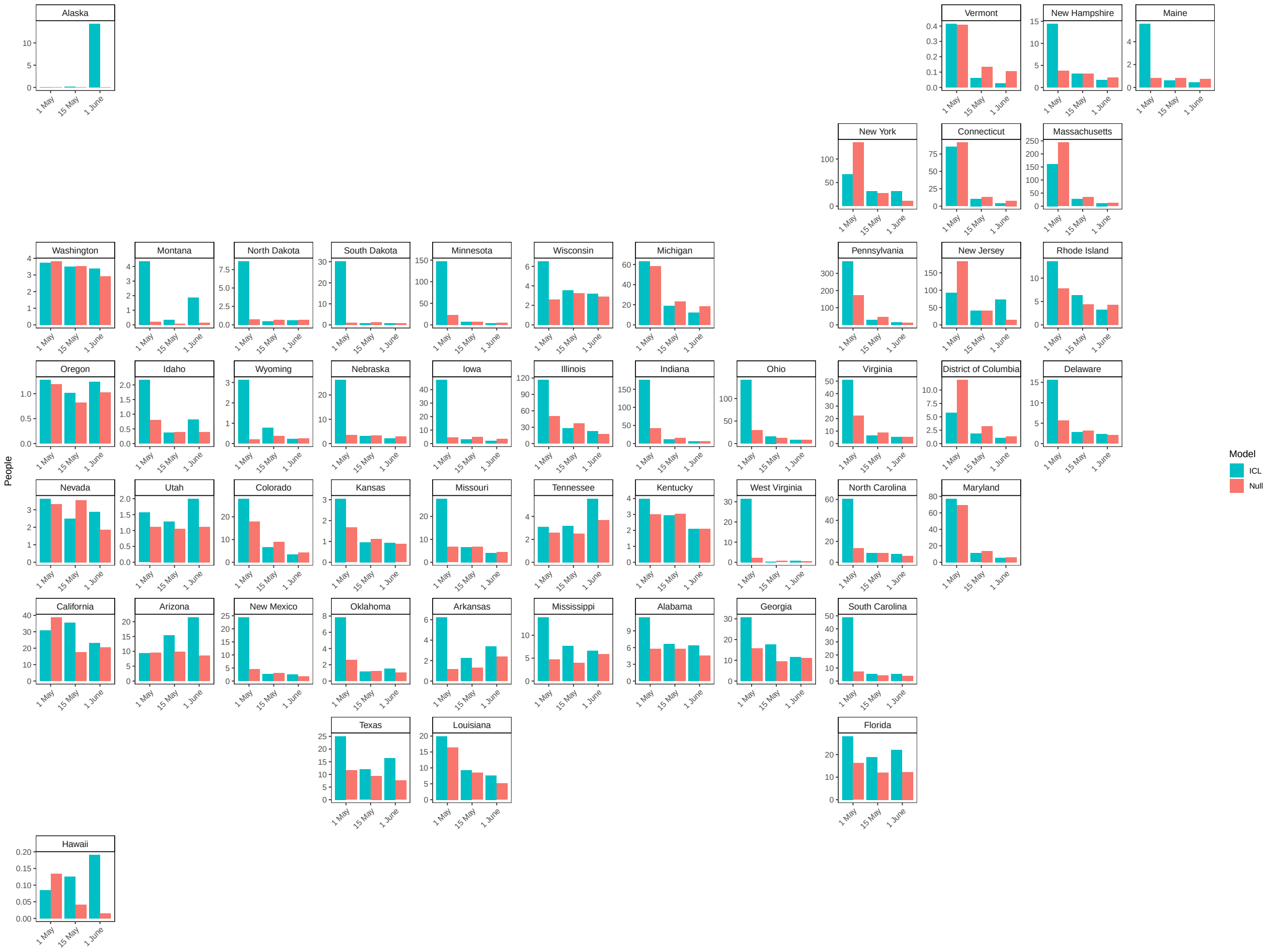

Supplementary Figure 9: **State level 21 day forecast CRPS estimates for three models fit until 1 May, 15 May and 1 June.** The blue ICL model refers to our model and the coral bars refer to the null model.

The mean coverage of the 50% credible interval is

$$\frac{1}{L}\sum_{t=t_0+1}^{t_0+1+L} 1\left(y_{t,m} \in \left[p_{25}(\{\widehat{y}^s_{t,m}\}_{s=1}^S), p_{75}(\{\widehat{y}^s_{t,m}\}_{s=1}^S)\right]\right). \tag{4}$$

The coverage was calculated for each state individually and then the mean across all the 50 state and the District of Columbia was computed. Again these coverage probabilities were compared with the log-linear "null" model.

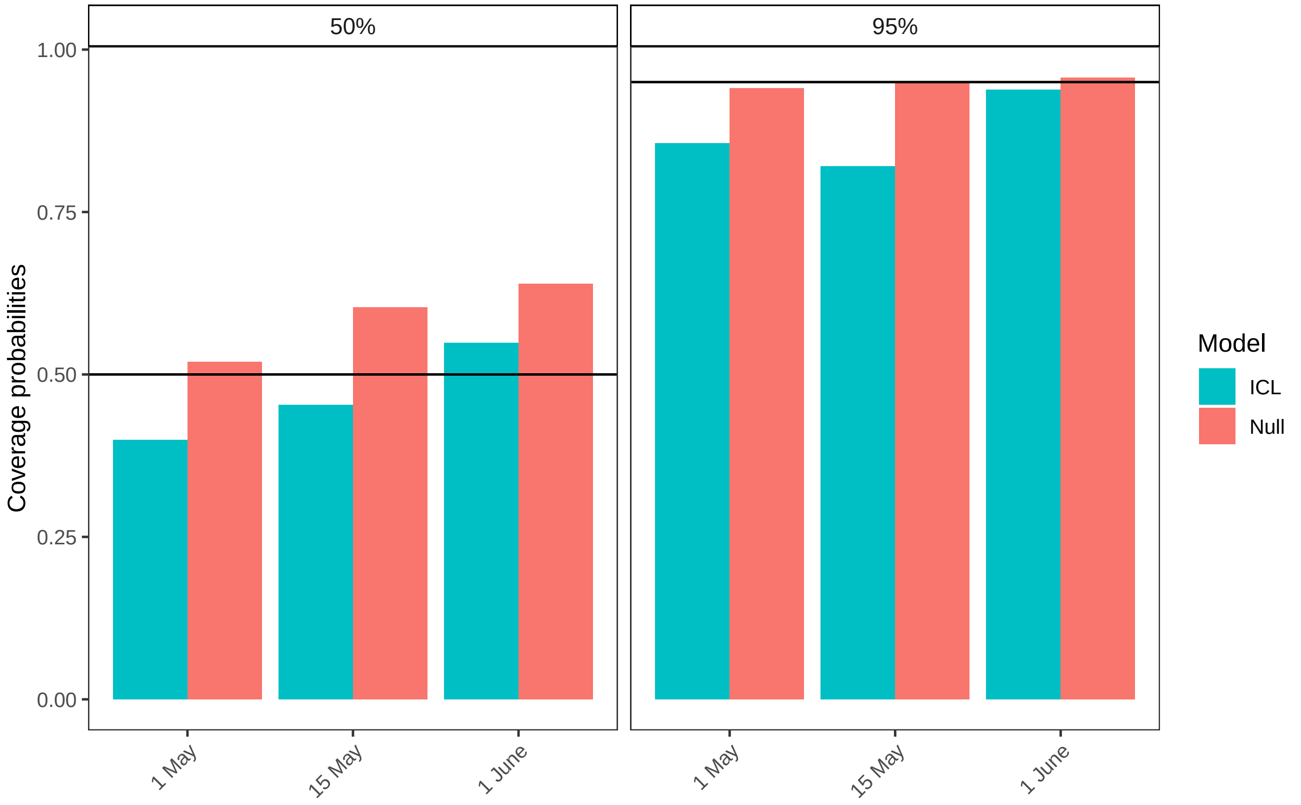

Supplementary Figure 10: **Coverage probabilities for 21 day forecasts for three models with data fit to 1 May, 15 May and 1 June.** The blue ICL model refers to our model and the coral bars refer to the null model.

These coverage results suggest that both the 95 and 50% credible intervals are well-calibrated for all the models but best for our model and the null model fit to the 1 June. We hypothesise this is because they include more in data in the forecast.

Supplementary Note 7 Mobility trends

In Supplementary Figure 11 we show the Google mobility trends [4] across the 50 states of the US and the District of Columbia. In our model, we use the time spent at one's residence and the number of visits to grocery stores, pharmacies, recreation centres, and workplaces. Our regions are based on US Census Divisions, modified to account for coordination between groups of state governments [5]. These trends are relative to a state-dependent baseline, which was calculated shortly before the COVID-19 epidemic. For example, a value of −20% in the average trend means that individuals, on average, are visiting 20% less shops, recreation places and workplaces

than before the epidemic. We overlay the timing of two major state-wide NPIs (stay at home and emergency decree) on Supplementary Figure 11 (see [6] for details). We note intuitive changes in mobility such as the spike on 11th and 12th April for Easter.

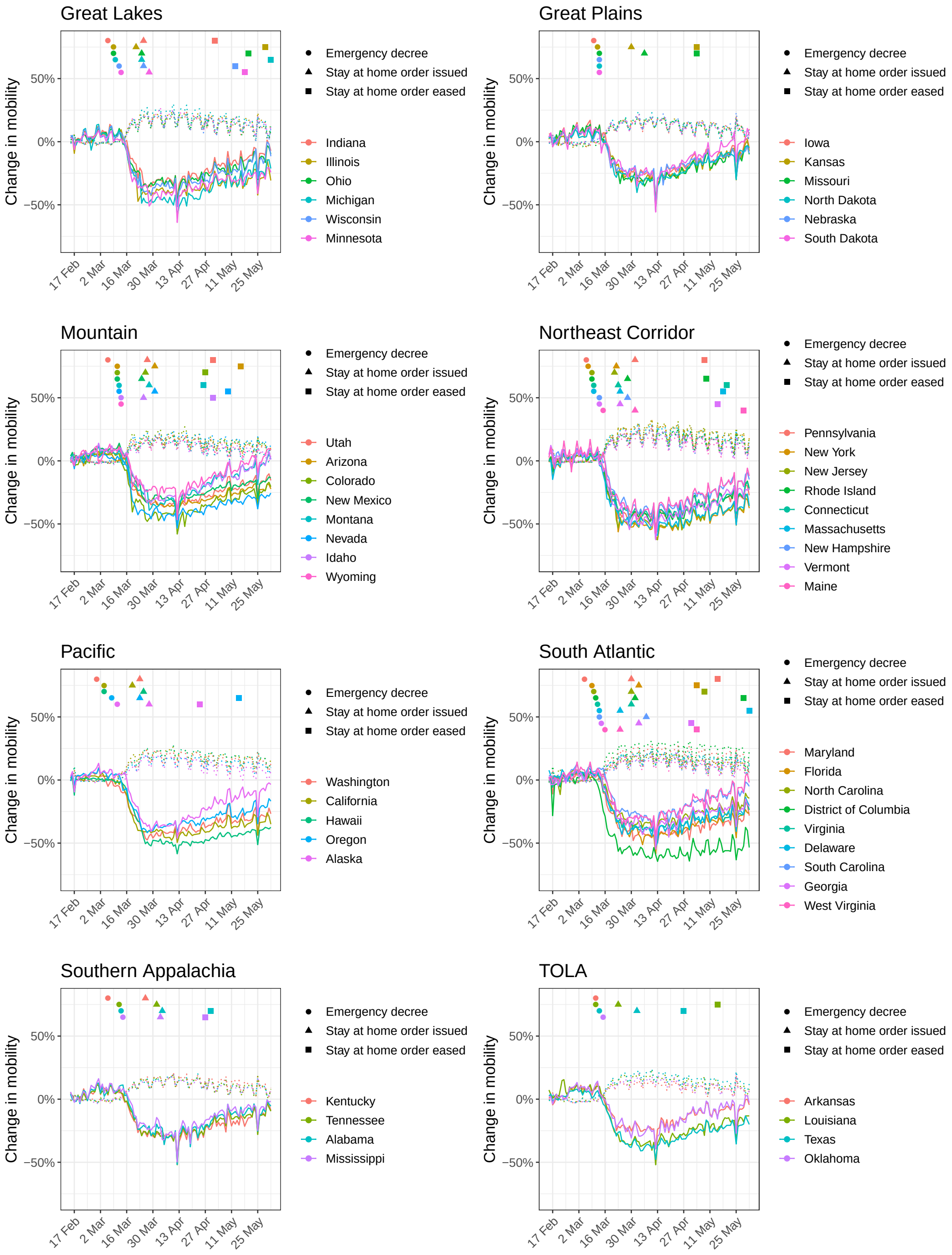

Supplementary Figure 11: **Comparison of mobility data from Google with government interventions for the 50 states and the District of Columbia.** The solid lines show average mobility (across categories "retail & recreation", "grocery & pharmacy", "workplaces") and the dotted lines show "residential". Intervention dates are indicated by shapes as shown in the legend; see Section 4.1 for more information about the interventions implemented. There is a strong correlation between the onset of interventions and reductions in mobility.

Supplementary Note 8 Mobility regression analysis

In Supplementary Figure 12 we regressed NPIs against average mobility. We parameterised NPIs as piece-wise constant functions that are zero when the intervention has not been implemented and one when it has. We evaluated the correlation between the predictions from the linear model and the actual average mobility. We also lagged the timing of interventions and investigate its impact on predicted correlation. We observed reduced correlation when lagging (forward and backwards) the timing of NPIs, which suggested an immediate impact on mobility.

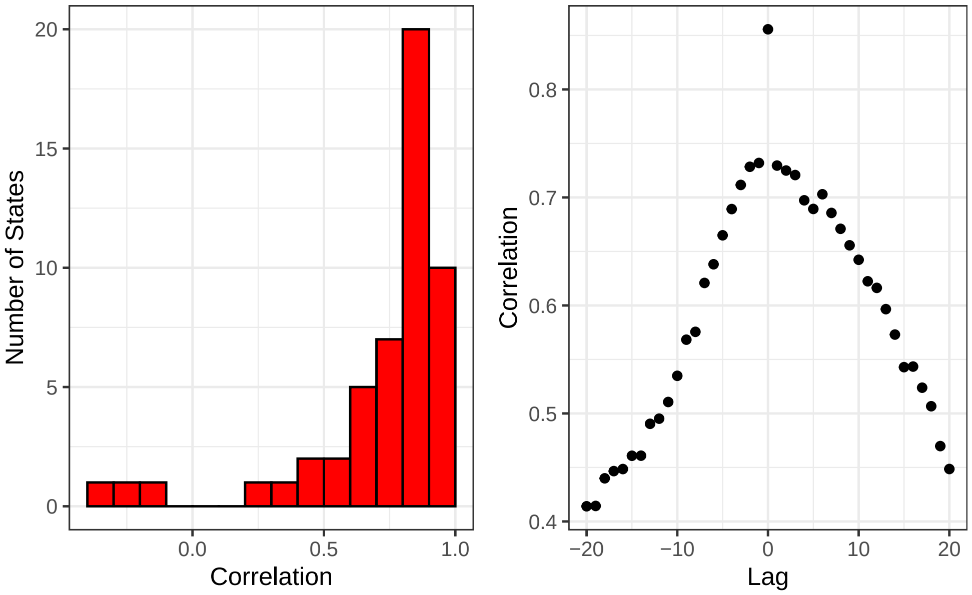

Supplementary Figure 12: **Mobility regression analysis.** The left figure is a histogram of the correlation between the predictions from the linear model and the average mobility. The right hand plot shows the effect of lagging the intervention timing on thee correlation.

Supplementary Note 9 Comparison of model attack rates with and without interventions in the model

We show in Supplementary Figure 13 that there is little difference between the attack rates for two models where we include the ”Stay at Home” intervention in our model as well and when we just use mobility. We, therefore, choose the mobility only model to limit the numbers of parameters we are fitting.

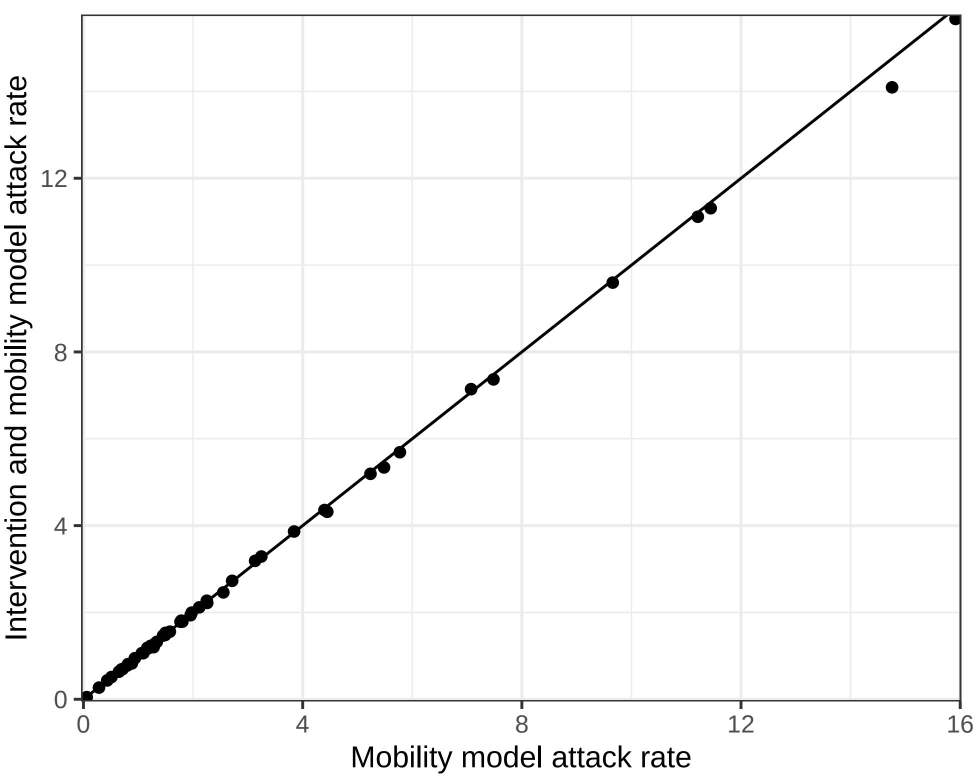

Supplementary Figure 13: **Comparison of model attack rates with and without interventions in the model**. The straight line has equation $y = x$.

Supplementary Note 10   State-specific weekly effects

Our model includes a state-specific weekly effect $\epsilon_{w,m}$ (see equations 12 and 14) for each week w in the epidemic period for a state. As described in Section 4, we assign an autoregressive process with mean 0 as prior to this effect. Supplementary Figure 14 shows the posterior of this effect on the same scale as in Figure 3, that is, the percent reduction in R_t with mobility variables held constant[1]. Values above 0 have the interpretation that the state-specific weekly effect lowers the reproduction number $R_{t,m}$, i.e. transmission for week t and state m is lower than what is explained by the mobility covariates.

[1]Draws from the posterior are transformed with $1 - f(-\epsilon_{m,w_m(t)})$, where $f(x) = 2\exp(x)/(1+\exp(x))$ is twice the inverse logit function.

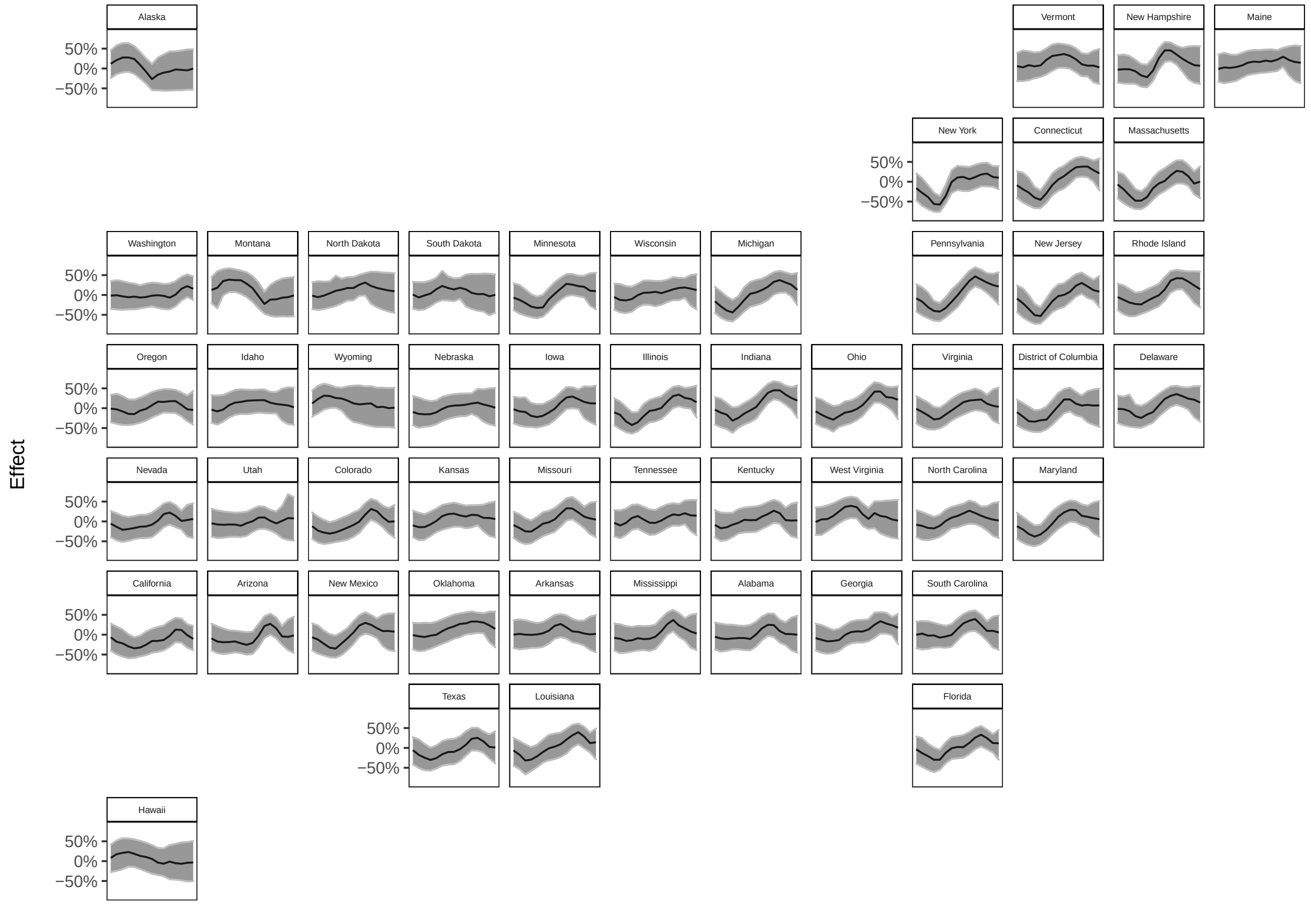

Supplementary Figure 14: **Percent reduction in R_t due to the weekly, state-level autoregressive effect.**

In Supplementary Figure 15, we compare the different terms contributing to R_t, as explained by equation 12, for three example states. We choose New York, Montana and Washington for our vignettes because the autoregressive (purple) trend behave differently in these states. In New York, the autoregressive term increases R_t before lockdown, which we hypothesise corresponds to importations and new seeding events from Europe driving transmission. Then the autoregressive term decreases as social distancing, mask wearing and handwashing are implemented along side behavioural changes. In contrast, the autoregressive term reduces R_t at the start in Montana and the mobility trends remain very flat, adding some weight to the hypothesis of importations. This could reflect the low density of that state and the correspondingly long distances between where people live, along with behavioural changes. The autoregressive term remains mostly constant in Washington, in line with the states early and effective epidemic response, and suggests that mobility is sufficient to capture the behaviour there.

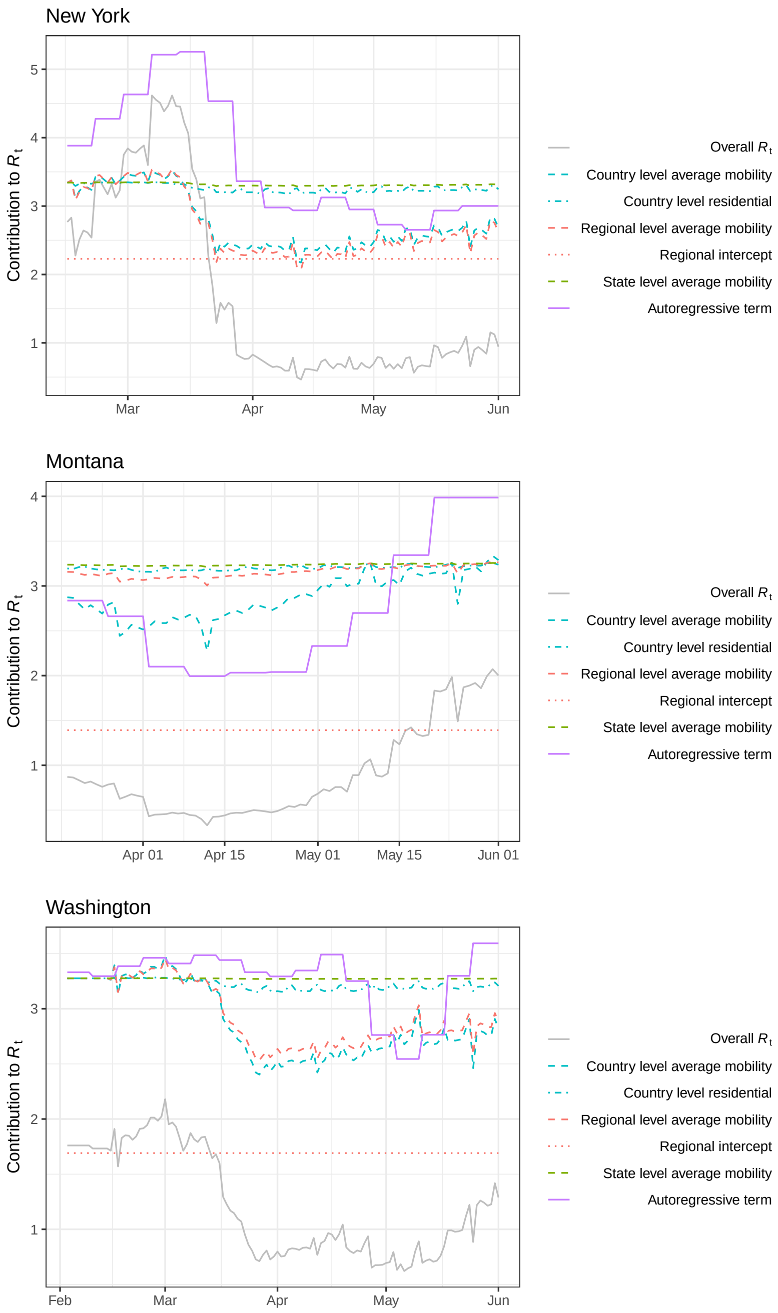

Supplementary Figure 15: **Comparison of different contributors to R_t for New York, Montana and Washington.** Here we consider all the contributions given in equation 12 - they grey solid line shows the posterior mean estimate of R_t and the coloured lines show the mean contributions from the other terms. We do not present confidence intervals here to avoid confusion. The colour denotes pooling level and the line type the mobility type where appropriate. Note the inverse logit transformation means these terms are not additive and each line, apart from the overall trend, is calculated by assuming all the other terms in equation 12 are zero.

Supplementary Note 11 Comparison of R_t estimations

We compare our R_t estimates on 01 June 2020 with estimates made by rt.live [7]. Overall, our estimates were weakly correlated ($\rho = 0.42$) with both of us estimating $R_t > 1$ in 23 states (red points) including Montana and

Alaska. However, the rt.live estimates are slightly more pessimistic because they predict $R_t > 1$ in 10 states where we predict $R_t < 1$ (blue points). In contrast we predict $R_t > 1$ in 5 states where they predict $R_t < 1$ (green points).

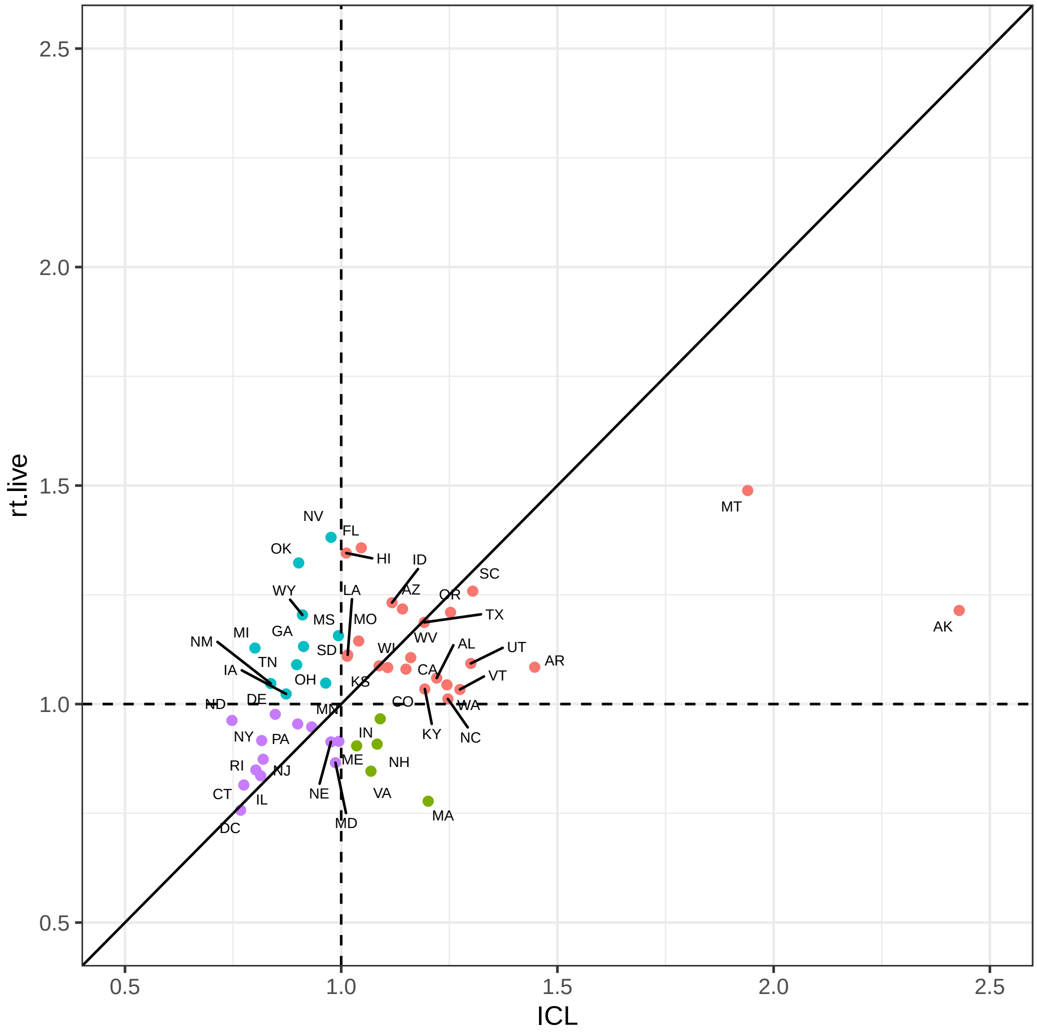

Supplementary Figure 16: **Comparison of our and rt.live [7] R_t estimates.** Colours are used to indicate groupings if each model predicted $R_t > 1$. Standard 2 letter state codes are used as labels- Alabama AL, Alaska AK, Arizona AZ, Arkansas AR, California CA, Colorado CO, District of Columbia DC, Connecticut CT, Delaware DE, Florida FL, Georgia GA, Hawaii HI, Idaho ID, Illinois IL, Indiana IN, Iowa IA, Kansas KS, Kentucky KY, Louisiana LA, Maine ME, Maryland MD, Massachusetts MA, Michigan MI, Minnesota MN, Mississippi MS, Missouri MO, Montana MT, Nebraska NE, Nevada NV, New Hampshire NH, New Jersey NJ, New Mexico NM, New York NY, North Carolina NC, North Dakota ND, Ohio OH, Oklahoma OK, Oregon OR, Pennsylvania PA, Rhode Island RI, South Carolina SC, South Dakota SD, Tennessee TN, Texas TX, Utah UT, Vermont VT, Virginia VA, Washington WA, West Virginia WV, Wisconsin WI and Wyoming WY.

Supplementary Note 12 Median absolute percent error

We compared the magnitude of our cumulative death median absolute percent error (MAPE) estimates from our forecasts from 1 June given in Supplementary Table 1 with global COVID-19 forecasts found in Friedman et

al. [8]. This is not a parameter we explicitly wanted to forecast but enabled us to compare with other models in literature. Friedman et al.'s paper included both SEIR type models (IHME – MS SEIR [9], Youyang Gu [10], MIT - DELPHI [11] and Imperial-LMIC [12]) and dynamic growth rate models (LANL-Growthrate [13]). The forecasts in Friedman et al. [8] were not all fit to the same date range, but to some point in June and are only presented for the whole of the US and not to each state specifically. Unlike the models compared by Friedman et al., the MAPE of our cumulative death forecasts did not increase significantly over time. Our 3 week median cumulative death MAPE across all states (9.9%) was similar to the US estimate from Friedman et al. (4.1-8.6 excluding the Imperial-LMIC model which was not calibrated for high income settings), but the magnitude was slightly higher in our forecasts for some states, in particular Alaska and Wyoming, which were the last two states to reach 10 cumulative deaths and so had the least data in our models.

Supplementary Table 1: **State level 1 week, 2 week and 3 week median absolute percentage error (MAPE) from our cumulative death forecasts fitted until 1 June.**

State	1 week	2 weeks	3 weeks
Alabama	7.8%	8.1%	9.1%
Alaska	75.0%	66.7%	50.0%
Arizona	9.1%	13.5%	17.4%
Arkansas	9.7%	10.4%	13.7%
California	7.4%	8.4%	10.0%
Colorado	7.1%	7.1%	7.1%
Connecticut	7.4%	7.4%	7.4%
Delaware	8.3%	8.3%	8.3%
District of Columbia	8.6%	8.3%	8.4%
Florida	8.5%	10.3%	12.9%
Georgia	6.6%	8.8%	10.2%
Hawaii	29.4%	29.4%	29.4%
Idaho	15.5%	13.6%	14.4%
Illinois	8.4%	8.9%	9.9%
Indiana	8.6%	9.4%	10.8%
Iowa	8.6%	8.2%	7.9%
Kansas	11.7%	11.4%	11.2%
Kentucky	8.1%	8.0%	7.9%
Louisiana	7.7%	7.7%	8.1%
Maine	13.1%	11.9%	11.8%
Maryland	7.6%	7.7%	7.9%
Massachusetts	7.6%	7.6%	7.3%
Michigan	7.8%	7.7%	7.7%
Minnesota	8.9%	9.8%	9.7%
Mississippi	9.3%	9.4%	11.7%
Missouri	7.6%	8.2%	10.3%
Montana	33.3%	31.6%	33.3%
Nebraska	11.1%	10.4%	10.4%
Nevada	9.0%	8.8%	8.9%
New Hampshire	11.5%	15.3%	16.2%
New Jersey	8.1%	8.0%	8.0%
New Mexico	10.5%	12.7%	14.5%
New York	7.8%	7.9%	7.9%
North Carolina	8.2%	8.0%	8.2%
North Dakota	13.9%	13.5%	13.0%
Ohio	7.5%	8.2%	8.9%
Oklahoma	8.6%	8.3%	8.1%
Oregon	9.8%	11.1%	12.0%
Pennsylvania	8.1%	8.9%	9.4%
Rhode Island	8.3%	8.8%	10.6%
South Carolina	8.4%	8.6%	9.4%
South Dakota	17.2%	13.5%	13.8%
Tennessee	8.3%	8.0%	9.4%
Texas	6.9%	7.3%	9.2%
Utah	11.6%	10.8%	11.7%
Vermont	16.1%	16.1%	15.8%
Virginia	7.5%	7.7%	8.6%
Washington	7.3%	7.6%	8.3%
West Virginia	14.3%	13.6%	15.7%
Wisconsin	8.3%	8.2%	8.4%
Wyoming	33.3%	31.2%	33.3%
**Median**	8.5%	8.8%	9.9%

Supplementary Note 13 Sensitivity analysis to infection fatality ratio

Geographic-specific contact surveys are important for calculating the weighted IFR values according to the methods in [14, 15]. There is no large-scale cross-generational contact survey, similar to the polymod survey [16], implemented in the USA. Therefore, it was important to understand if the model was robust to changes in the underlying contact survey. We calculated the IFRs using three different contact matrices: UK, France and Netherlands. We believe that the USA is culturally closest to that UK out of those countries we had contact matrices for, but also considered France where we saw the greatest mixing of the elderly and the Netherlands which showed the *average* behaviour of the European studies used in [15]. We found that the IFR, calculated for each state using the three contact matrices, lay within the posterior of IFR in our model (Supplementary Figure 17). We also noted that our results remained approximately constant when using the IFR calculated from the three different contact matrices as the mean of the prior IFR in out model, see Section 4.

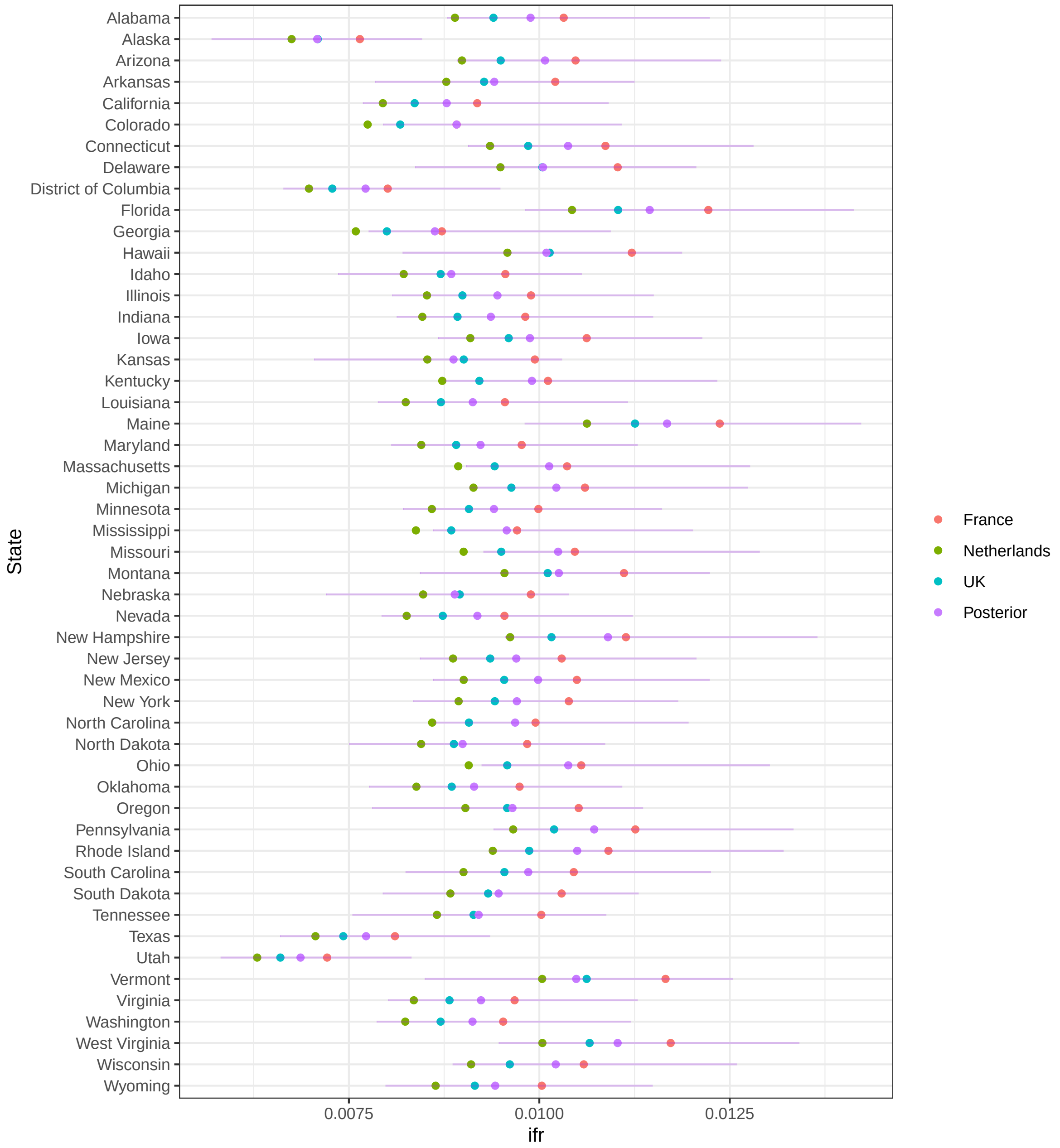

Supplementary Figure 17: **Sensitivity analysis for IFR**. The red, green and blue dots show the IFR point estimates calculated according to [14, 15] using the French, Dutch and and UK contact matrices respectively. The purple dot shows the mean of our posterior estimates for the IFR run using the UK contact matrix estimate (central measure of the error bars) and the purple error bars shows the 95% credible intervals of the distribution. The sample size n = 105,006 deaths across the 50 states and the District of Columbia up until 1 June and 479,422 cases from 11 May to 1 June.

Since we are using the same contact matrix across all the states, the differences in IFR are due to the population demographics and not due to differential contacts. The low IFR in Texas and Utah reflects the younger population there whereas the higher IFR in Florida and Maine is due to the older population. This is a limitation of our methods.

Supplementary References

1. U.S. Census Bureau. *Cartographic Boundary Files - Shapefile* https://www.census.gov/geographies/mapping-files/time-series/geo/carto-boundary-file.html. 2020.
2. Gneiting, T. & Raftery, A. E. Strictly Proper Scoring Rules, Prediction, and Estimation. *Journal of the American Statistical Association* **102,** 359–378. eprint: https://doi.org/10.1198/016214506000001437. https://doi.org/10.1198/016214506000001437 (2007).
3. Marchant, R, Samia, N. I., Rosen, O, Tanner, M. A. & Cripps, S. *Learning as We Go: An Examination of the Statistical Accuracy of COVID19 Daily Death Count Predictions* 2020. arXiv:2004.04734.
4. Aktay, A *et al. Google COVID-19 Community Mobility Reports: Anonymization Process Description (version 1.0)* 2020.
5. Reston, M., Sgueglia, K. & Mossburg, C. *Governors on East and West coasts form pacts to decide when to reopen economies* 2020. https://edition.cnn.com/2020/04/13/politics/states-band-together-reopening-plans/index.html.
6. Fullman, N *et al. State-level social distancing policies in response to COVID-19 in the US* 2020. http://www.covid19statepolicy.org.
7. Systrom, K., Vladek, T. & Krieger, M. *Project Title* https://github.com/rtcovidlive/covid-model. 2020.
8. Friedman, J., Liu, P., Gakidou, E. & IHME COVID-19 Model Comparison Team. Predictive performance of international COVID-19 mortality forecasting models. *medRxiv* (2020).
9. IHME. *COVID-19 Projections* https://covid19.healthdata.org/global. 2020.
10. Gu, Y. *COVID-19 Projections Using Machine Learning* https://github.com/youyanggu/covid19_projections. 2020.
11. Li, M. L. *et al.* Forecasting COVID-19 and Analyzing the Effect of Government Interventions. *medRxiv* (2020).
12. Imperial College. *Future scenarios of the healthcare burden of COVID-19 in Low- or Middle-Income Countries* https://github.com/mrc-ide/global-lmic-reports. 2020.
13. Los Alamos National Laboratory. *LANL COVID-19 Cases and Deaths Forecasts* https://covid-19.bsvgateway.org. 2020.
14. Verity, R *et al.* Estimates of the severity of COVID-19 disease. *Lancet Infect Dis* (2020).
15. Walker, P *et al.* The impact of COVID-19 and strategies for mitigation and suppression in low- and middle-income countries. *Science.* https://www.imperial.ac.uk/mrc-global-infectious-disease-analysis/news--wuhan-coronavirus/ (2020).
16. Mossong, J *et al.* Social Contacts and Mixing Patterns Relevant to the Spread of Infectious Diseases. *PLOS Medicine* **5,** 1–1. https://doi.org/10.1371/journal.pmed.0050074 (Mar. 2008).
